# Molecular Signature of Subtypes of Non-Small-Cell Lung Cancer by Large-Scale Transcriptional Profiling: Identification of Key Modules and Genes by Weighted Gene Co-Expression Network Analysis (WGCNA)

**DOI:** 10.3390/cancers12010037

**Published:** 2019-12-21

**Authors:** Magdalena Niemira, Francois Collin, Anna Szalkowska, Agnieszka Bielska, Karolina Chwialkowska, Joanna Reszec, Jacek Niklinski, Miroslaw Kwasniewski, Adam Kretowski

**Affiliations:** 1 Clinical Research Centre, Medical University of Bialystok, 15-276 Bialystok, Poland; anna.szalkowska@umb.edu.pl (A.S.); agnieszka.bielska@umb.edu.pl (A.B.); adamkretowski@wp.pl (A.K.); 2 Centre for Bioinformatics and Data Analysis, Medical University of Bialystok, 15-276 Bialystok, Poland; francois.collin@umb.edu.pl (F.C.); karolina.chwialkowska@umb.edu.pl (K.C.); miroslaw.kwasniewski@umb.edu.pl (M.K.); 3 Department of Medical Pathomorphology, Medical University of Bialystok, 15-276 Bialystok, Poland; joanna.reszec@umb.edu.pl; 4 Department of Clinical Molecular Biology, Medical University of Bialystok, 15-276 Bialystok, Poland; jacek.niklinski@umb.edu.pl; 5 Department of Endocrinology, Diabetology and Internal Medicine, Medical University of Bialystok, 15-276 Bialystok, Poland

**Keywords:** non-small-cell lung cancer, squamous cell lung cancer, adenocarcinoma, transcriptomic profiling, next-generation sequencing, WGCNA

## Abstract

Non-small-cell lung cancer (NSCLC) represents a heterogeneous group of malignancies consisting essentially of adenocarcinoma (ADC) and squamous cell carcinoma (SCC). Although the diagnosis and treatment of ADC and SCC have been greatly improved in recent decades, there is still an urgent need to identify accurate transcriptome profile associated with the histological subtypes of NSCLC. The present study aims to identify the key dysregulated pathways and genes involved in the development of lung ADC and SCC and to relate them with the clinical traits. The transcriptional changes between tumour and normal lung tissues were investigated by RNA-seq. Gene ontology (GO), canonical pathways analysis with the prediction of upstream regulators, and weighted gene co-expression network analysis (WGCNA) to identify co-expressed modules and hub genes were used to explore the biological functions of the identified dysregulated genes. It was indicated that specific gene signatures differed significantly between ADC and SCC related to the distinct pathways. Of identified modules, four and two modules were the most related to clinical features in ADC and SCC, respectively. *CTLA4*, *MZB1*, *NIP7*, and *BUB1B* in ADC, as well as *GNG11* and *CCNB2* in SCC, are novel top hub genes in modules associated with tumour size, SUV_max_, and recurrence-free survival. Our research provides a more effective understanding of the importance of biological pathways and the relationships between major genes in NSCLC in the perspective of searching for new molecular targets.

## 1. Introduction

Lung cancer is the second commonly diagnosed cancer in both men and women, as well as the leading cause of cancer-related mortality in the world. According to the American Cancer Society, approximately 228,150 new cases of lung cancer were diagnosed, and over 142,000 deaths were registered in the United States in 2019 alone [1]. Lung cancer is classified as either small-cell lung carcinoma (SCLC) or non-small-cell lung carcinoma (NSCLC). NSCLC is diagnosed in four out of five cases of lung cancer. The main subtypes of NSCLC are squamous cell carcinoma (SCC), adenocarcinoma (ADC), and large-cell carcinoma (LCC) [2]. Unfortunately, despite the introduction of new treatment options in recent years, the survival rates of patients with NSCLC remain unsatisfactory. Currently, the gold standard is the therapy of NSCLC based on histopathological features. However, progression, metastasis susceptibility, and potential response to treatment cannot be fully predicted based on histopathological observation. Furthermore, even if NSCLC patients have similar clinical characteristics and have tumours at a similar stage, they may experience different clinical outcomes [3]. Recent studies displayed that the histologic types of lung cancer differ in their molecular features, such as mutational signatures, DNA copy number, and gene expression [4,5,6,7]. Due to this fact, the molecular genetic classification of cancers is attracting widespread interest nowadays. The next-generation sequencing (NGS) plays a key role in cancer profiling. RNA-seq avoids the limitation of microarray analysis and allows enhanced detection of low-abundance transcripts. However, the attempt to explain the complex relationship between genes is still a challenge [8,9]. The differential expression analysis allows considering each gene individually and does not account for the connection between genes. Since it is known that biological processes are based on the numerous interactions between many cellular components, gene network analysis could provide precious information on cancer pathogenesis [10].

The Weighted Correlation Network Analysis (WGCNA) [11,12] corresponds to a data reduction method and unsupervised classification method. It simplifies the interpretation of thousands of gene responses to a dozen of synthetic groups (or modules) of genes. The net establishes connections between genes-genes are connected if their expression is correlated. Genes can be more or less intensively connected depending on the value of the correlation (the weights). The connectivity between genes is then interpreted into a distance, and the distance is used to group genes into modules. This is how a high number of genes can be reduced into a small number of clusters whose expression is quantified by the Eigengenes (first principal component within the module). It relies on the assumption that highly correlated genes within a module are involved in common biological processes. For example, Tian et al. [13] used WGCNA to identify two gene co-expression modules involved in the process of lung SCC metastasis and suggested that *CFTR*, *SCTR*, and *FIGF* genes could be used as a potential prognostic biomarker. Using WGCNA in the studies of progression and prognosis of breast cancer indicated the relevant correlation of one module of genes with patient survival time. Moreover, the eight candidate biomarkers of the pathogenesis of breast cancer have been identified [14]. Yin et al. [15] found through WGCNA that five high degree hub genes may play a key role in the hepatocellular carcinoma (HCC) progression. Additional studies using WGCNA have shown that *SKA1*, *ERCC6L*, and *GTSE-1* may be potential diagnostic markers for renal cell carcinoma [16]. Furthermore, WGCNA has been also applied for RNA-seq and miRNA-seq data of colon adenocarcinoma (CAC). These studies defined two gene modules and one miRNA module correlated with the pathological stage of CAC, as well as the putative representative biomarkers related to prognosis were defined [17]. In addition, Nakamura et al. [18] showed in proteomics that data analysis using WGCNA was notably effective to group proteins into modules and to identify of hub proteins, which played key roles in small-cell lung carcinoma and large-cell neuroendocrine lung carcinoma.

The broad goal of the current study was to identify transcriptome profile associated with the histological subtypes of NSCLC in a well-characterised group of patients from MOBIT project using RNA-seq. The Molecular Biomarkers for Individualized Therapy (MOBIT) [19] is an ambitious study targeting the development of personalised diagnostics based on high-quality biobanking and integrated analysis of ‘omics’ and imaging PET/MRI (Positron Emission Tomography-Magnetic Resonance Imaging) analysis, including the phenotypic, clinical, and epidemiological traits. As part of the project MOBIT, biospecimens of lung tumour and normal tissues were collected under strictly monitored conditions. MOBIT’s unique approach to the acquisition of tumour samples considers the following parameters: time of anaesthesia start, time of surgery start, time of resection start, time of vessel ligation, and time of resection end. The above parameters affect the molecular reality of individual tumours. The tissue ischemia time affects gene and protein expression patterns within minutes following surgical tumour excision [20]. The know-how of managing the oncology biobank was obtained from a scientific partner-Indivumed GmbH [21,22]. Differential expression analysis was performed to detect differentially expressed genes (DEGs) in ADC and SCC tissues vs. normal tissues. In addition, we applied the weighted gene co-expression network analysis (WGCNA) to find highly correlated gene modules with clinical traits and to identify the key genes in selected modules with the highest connectivity in the module in question.

## 2. Results

### 2.1. Baseline Characteristic

During the study program from 2015–2016, 114 patients with NSCLC were enrolled in the MOBIT project and underwent surgical tumour resection [19]. The mean age at diagnosis was 65.4 years, and 80 patients (70.2%) were male. One hundred and seven patients were smokers, with the average number of pack years was 39.6, whereas seven patients never smoked. Fourty-five (39.5%) had adenocarcinoma, and 69 (60.5%) had squamous cell carcinoma. Stage I disease was identified in 39 patients, stage II in 52 patients, stage III in 17 patients, and stage IV in six patients (Table 1). During lung tumour excision procedures, one to three fragments of lung tissues were collected from each patient and classified by pathologists as normal (Tn) and tumour (Tp, peripheral and/or Tc, central).

### 2.2. Gene Expression Profile Data in NSCLC Subtypes

**Figure 1:**
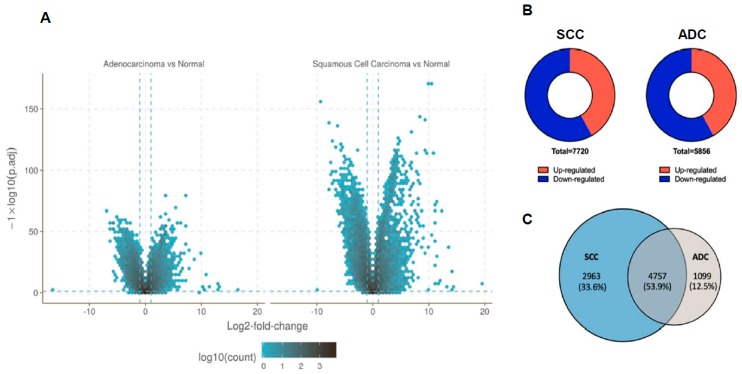
Differential gene expression in squamous cell carcinoma (SCC) and adenocarcinoma (ADC). (**A**) Volcano plot of differentially expressed genes (DEGs) in each subtype of non-small-cell lung carcinoma (NSCLC). The horizontal line at false discovery rate (FDR) = 0.05; vertical line at |log2FC| = 1; (**B**) Pie charts represent the numbers of genes found to be upregulated or downregulated. (**C**) Venn diagram shows overlap between differentially expressed genes in SCC and ADC. The numbers of DEGs include both up- and down-regulated genes in tumour tissue compared to normal tissue.

A total of 228 samples, 114 tumour and 114 normal tissues, were included in the differential expression analysis with the count depth of higher than 15 million reads. Analysis was done in the software R along with two packages, edgeR [23] and DESeq2 [24]. Due to the large size of the tested genes, raw p values were adjusted according to Benjamini and Hochberg’s method. The selection criteria were strengthened with a threshold of FDR ≤ 0.05 and |log2FC| ≥ 1. Volcano plots were plotted to illustrate the distribution of each gene according to the log2FC and FDR (Figure 1A). These stringent criteria generated a list of 7720 genes differentially expressed in SCC in comparison to normal tissue, with 3233 up-regulated and 4487 down-regulated genes; 5856 of genes were significantly differentially expressed in ADC in comparison to normal tissue, with 2452 up-regulated and 3404 down-regulated genes (Figure 1B,C). Interestingly, among the up-regulated and down-regulated genes, 4757 (53.9%) were overlapping between SCC and ADC, while 4062 (46.1%) were specific to SCC or ADC.

**Figure 2:**
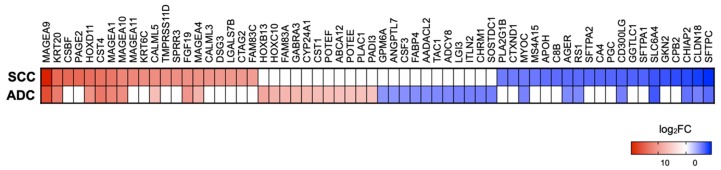
Top 20 genes up- and downregulated in SCC and ADC tissue compared to normal tissue (FDR ≤ 0.01). In the heat map colour indicated log fold change, shades of blue represent log2FC < −1, and shades of red represent log2FC > 1 and missing values are in white. FC, fold change.

The top 20 genes with the greatest upregulation and downregulation in cancer tissues are summarised in Figure 2. The common upregulated genes in SCC and ADC included *MAGEA9*, *KRT20*, *HOXD11*, *CST4*, *MAGEA1*, *MAGEA10*, *MAGEA4*, *FGF19*, and *CALM5*. These genes are mainly associated with extracellular matrix and proliferation, promotion of tumorigenesis, and apoptotic processes [25,26,27]. Interestingly, as many as four top up-regulated genes in lung cancer tissue were homologous genes from the MAGE-A family. It should be noted that the remaining genes in ADC from the top 20 were also up-regulated in SCC but at a lower level. Nevertheless, we found that *CSBF*, *PAGE2*, *CALM3*, *DSG3*, *CTAG2*, and *FAM83C* were up-regulated only in SCC.

The common downregulated genes in SCC and ADC included *SFTPC*, *CLDN18*, *CHIAP2*, *SLC6A4*, *CD300LG*, *RS1*, *AGER*, and *MYOC*. Majority of these genes are mainly involved in differentiation, cell death, cell cycle progression, regulation of immune response, migration, and lung adenocarcinoma formation [28,29,30]. The most down-regulated gene in both subtypes is *SFTPC*, encoding surfactant protein c, a hydrophobic protein with the capacity of mitigating surface tension in the lung tissue [31]. We noticed that other top down-regulated genes in ADC were also down-regulated in SCC but at a lower level.

### 2.3. Gene Ontology and Canonical Pathway Analysis 

To identify biologic pathways, networks, and functional categories of differentially expressed genes, we used a combination of bioinformatic tools: the Ingenuity Pathway Analysis (IPA) (QIAGEN Inc., https://qiagenbioinformatics.com/products/ingenuity-pathway-analysis), the gene ontology (GO) enrichment analysis-biological processes (BP) by BiNGO tool [32] as plugin for Cytoscape version 3.7.2 (http://cytoscape.org/), and a novel tool to provide a comprehensive gene list annotation based on over 40 independent knowledge bases, Metascape (http://metascape.org) [33].

**Figure 3:**
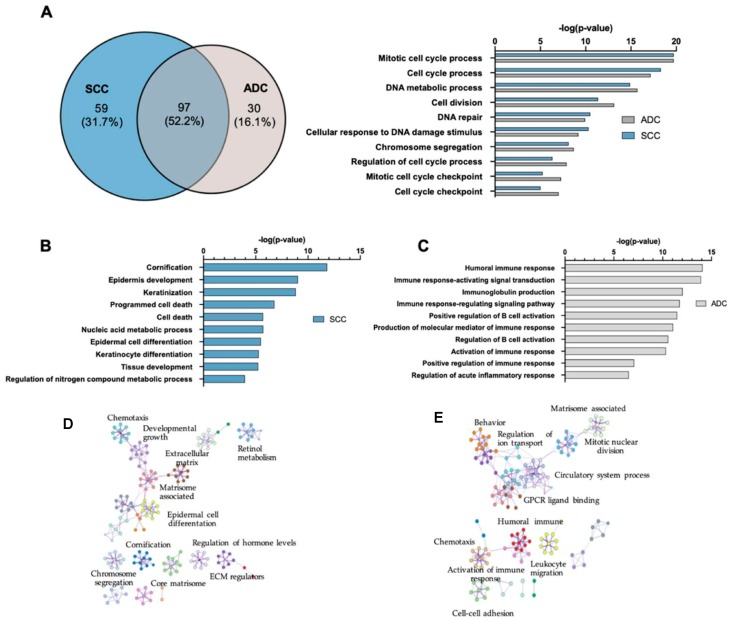
Gene ontology (GO)-terms analysis. (**A**) Venn diagram showing the number of GO-biological process (BP) differentially expressed (FDR ≤ 0.01) in squamous cell lung carcinoma (SCC) and adenocarcinoma (ADC) and the number of overlapping GO-terms between this both subtypes of NSCLC. (**B**) Top 10 GO-terms identified only in SCC vs. normal tissue. (**C**) Top 10 GO-terms identified only in ADC vs. normal tissue. (**D**) The network of enriched terms coloured by cluster identified only in SCC vs. normal tissue using Metascape tool. (**E**) The network of enriched terms coloured by cluster identified only in ADC vs. normal tissue using Metascape tool.

Among all the GO-terms (BP), only 97 (52.2%) overlapped between SCC and ADC. GO-enrichment analysis revealed that among the DEGs were those involved in cell cycle regulation, proliferation, metabolic processes, and DNA repair (Figure 3A). These processes are usually disrupted in cancer and are potentially involved in the initiation and/or promotion of tumorigenesis. When we looked at the GO-terms in each subtype of NSCLC, we noticed that, in SCC, the deregulated processes (31.7%) were focused around cornification (FDR = 1.47 × 10^−12^), epidermis development (FDR = 9.11 × 10^−10^), keratinisation (1.49 × 10^−9^), and epidermal cell differentiation (3.2 × 10^−6^) (Figure 3B). In contrast, GO-terms in ADC (16.1%) were related with the humoral immune response (1.21 × 10^−17^), production of molecular mediator of immune response (1.83 × 10^−14^), activation of immune response (1.32 × 10^−13^), and positive regulation of immune response (2.98 × 10^−10^) (Figure 3C). The most significant pathways identified by Metascape in SCC included cornification, epidermal cell differentiation, chemotaxis, matrisome-associated, core matrisome, developmental growth, and ECM regulators (Figure 3D). Humoral immunity, activation of immune response, leukocyte migration, chemotaxis, cell-cell adhesion, matrisome-associated, and mitotic nuclear division were the most significant pathways in ADC tissues (Figure 3E).

**Figure 4:**
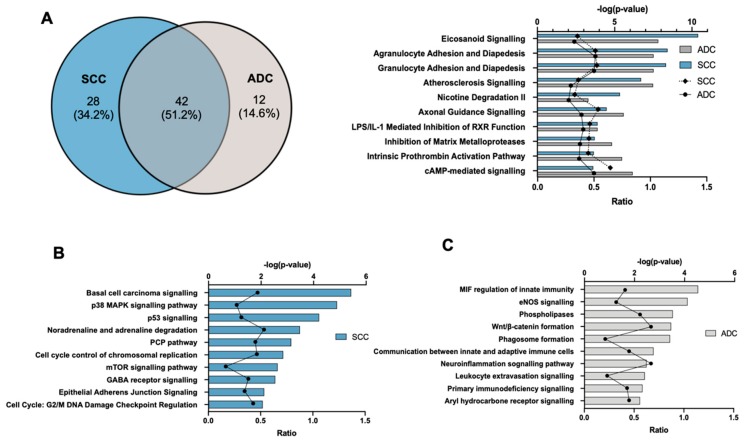
The most representative altered canonical pathways associated with SCC and ADC identified using Ingenuity Pathway Analysis (IPA). (**A**) Venn diagram showing the number of canonical pathways (FDR ≤ 0.01) in squamous cell lung carcinoma (SCC) and adenocarcinoma (ADC), and the overlapping of those pathways between these both subtypes of NSCLC. (**B**) Top 10 canonical pathways identified only in SCC vs. normal tissue. (**C**) Top 10 canonical pathways identified only in ADC vs. normal tissue. ‘Ratio’ refers to the number of molecules from the dataset that map to the pathway listed, divided by the total number of molecules that define the canonical pathway from within the IPA knowledge base (https://www.qiagenbioinformatics.com/products/ingenuity-pathway-analysis/).

Ingenuity core analysis identified 70 and 54 altered canonical pathways in SCC and ADC, respectively (FDR ≤ 0.01). The top 10 pathways of each subtype with a highly significant correlation, in comparison to the normal tissue, are listed in Figure 4A. Among them, 42 (51.2%) canonical pathways were common to both subtypes. Eicosanoid signalling, agranulocyte and granulocyte adhesion and diapedesis, atherosclerosis signalling, and axonal guidance signalling were the most significant changed pathways in both subtypes of NSCLC.

IPA analysis for all differentially expressed genes in SCC vs. normal tissue revealed basal cell carcinoma signalling (FDR = 3.72 × 10^−6^, ratio = 33/70), p38 MAPK signalling pathway (FDR = 1.29 × 10^−5^, ratio = 32/54), p53 signalling pathway (FDR = 7.59 × 10^−3^, ratio = 34/62), and noradrenaline degradation (FDR = 7.05 × 10^−4^, ratio=16/30) as the top canonical pathways, not disclosed in ADC (Figure 4B). Overall, these pathways and GO-terms results indicate that epithelial squamous cells transformation and excessive proliferation play an important role in SCC pathogenesis.

The top dysregulated canonical pathways detected in ADC were MIF regulation of innate immunity (FDR = 2.88 × 10^−5^, ratio = 16/42), eNOS signalling (FDR = 2.54 × 10^−3^, ratio = 16/42), phospholipases (FDR = 2.95 × 10^−3^, ratio 20/57), and Wnt/β-catenin formation (FDR = 3.46 × 10^−3^, ratio 47/170). Interestingly, most of the biological processes and pathways activated in ADC were related to tumour immune response (Figure 4C).

### 2.4. Upstream Transcription Regulators

**Figure 5:**
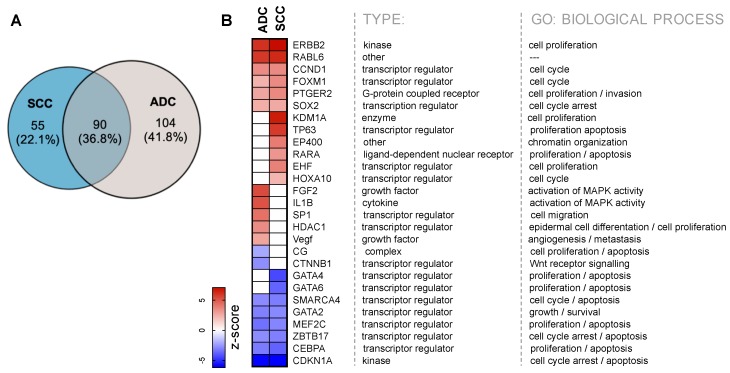
IPA-predicted top 20 upstream regulators of gene expression in SCC and ADC. (**A**) Venn diagram showing the number of upstream regulators (FDR ≤ 0.01) in squamous cell lung carcinoma (SCC) and the number of overlapping regulators between these both subtypes of NSCLC. (**B**) The heat map shows the predicted activation and inhibition states of categorised molecules in IPA as transcription regulators, growth factors, cytokines, kinases and others. Colours in heat map indicate z-scores.

The upstream regulator analysis tool is a function in IPA which may, using analysing linkage to DEGs through coordinated expression, identify assumed upstream regulators that have been observed experimentally to affect gene expression [34]. We identified the upstream transcriptional regulators with FDR ≤ 0.01 and the absolute activation z-score > 2 or <–2. Among activated or inhibited regulators, only 90 (36.8%) were overlapping between SCC and ADC (Figure 5A). As shown in Figure 5B, the majority of regulators were transcriptional regulators, growth factors, kinases, and enzymes, etc., which are related to cell growth, cell cycle, invasion, and apoptosis. *ERBB2*, with the highest activation z-score, encodes a member of the epidermal growth factor (EGF) receptor family of receptor tyrosine kinases, which is involved in the development of human malignancies [35,36]. *CDKN1A*, with the lowest z-score, encodes cyclin-dependent kinase inhibitor 1, which plays a major part in cell cycle arrest in response to DNA damage [37].

However, as expected, we observed groups of regulators specific to each NSCLC subtypes. Within the IPA-predicted top 20 upstream regulators, almost half of them were different in SCC and ADC. For the DEGs in SCC, IPA identified 145 activated or inhibited upstream regulators, among which there are regulators such as *RARA*, *KDM1A*, *HOXA10*, *EHF*, *EP400*, *GATA4*, *GATA6*, and *TP63* characteristic only for SCC. *KDM1A*, with the highest activation z-score, encodes lysine demethylase 1, which contributes to tumorigenesis in many types of cancer [38], while *TP63* encodes p63 protein which is a master regulator in epithelial development and has shown significance in cancer progression [39].

For the DEGs in ADC, IPA identified 194 activated or inhibited upstream regulators, among which there are regulators such as *FGF2*, *IL1B*, *SP1*, *HDAC1*, *VEGF*, *CG*, and *CTNNB1*. *FGF2*, with the highest z-score in ADC encodes the fibroblast growth factor 2, which is associated with multiple cancer pathogenesis process including cell proliferation, differentiation and metastasis [40]. *IL1B,* the second upstream regulator with the high z-score in ADC, encodes IL-1β which is one of the inflammatory cytokines, plays a key role in carcinogenesis and tumour progression [41]. The target molecules list for each upstream regulator is provided in Table S1.

### 2.5. Construction of Weighted Gene Co-Expression Network 

**Figure 6:**
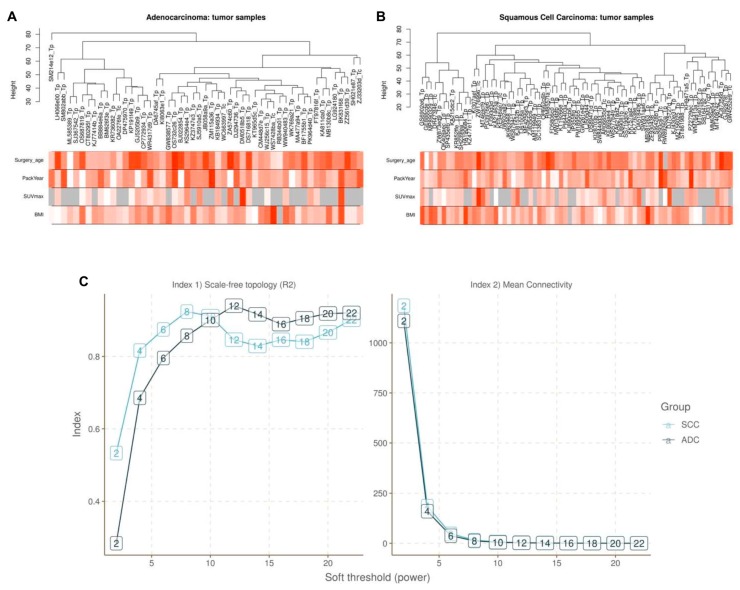
Cluster analysis of samples of (**A**) ADC and (**B**) SCC to detect outliers (white-to-red linear gradient colour associated with corresponding clinical variable, grey when missing data). (**C**) Determination of soft-thresholding power in weighted gene co-expression network analysis (WGCNA).

WGCNA was performed to construct co-expressed networks and identify co-expression modules. Hierarchical clustering of the samples was performed based on an euclidean distance computed on log10-transformed RNA-seq fractional counts (Figure 6A,B). Additionally, basic patient information was added below the resulting tree. The sample aggregation suggested by the resulting trees does not identify strong outliers, and the basic patient information does not suggest the inclusion of an atypical patient. The research of an adequate soft-threshold validating converged toward scale-free topology, pointing at values 10 and 7 for ADC and SCC, respectively (Figure 6C).

**Figure 7:**
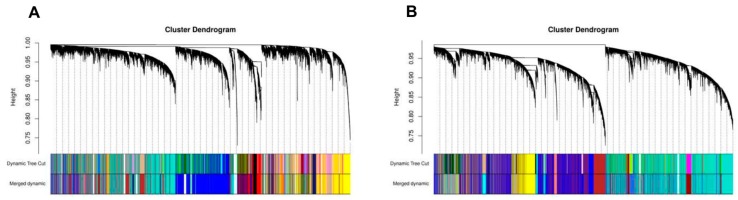
Hierarchical clustering dendrograms of identified co-expressed genes in modules in (**A**) ADC and (**B**) SCC lung cancer. Each coloured row represents a colour-coded module which contains a group of highly connected genes. A total of 32 and 15 modules was identified in ADC and SCC, respectively.

As shown in Figure 7, the first set of modules was obtained with Dynamic Tree Cut algorithm, then correlated modules (r > 0.75) were merged together (Merged dynamic); 32 and 15 modules were identified in ADC and SCC, respectively (Figure 7A,B).

**Figure 8:**
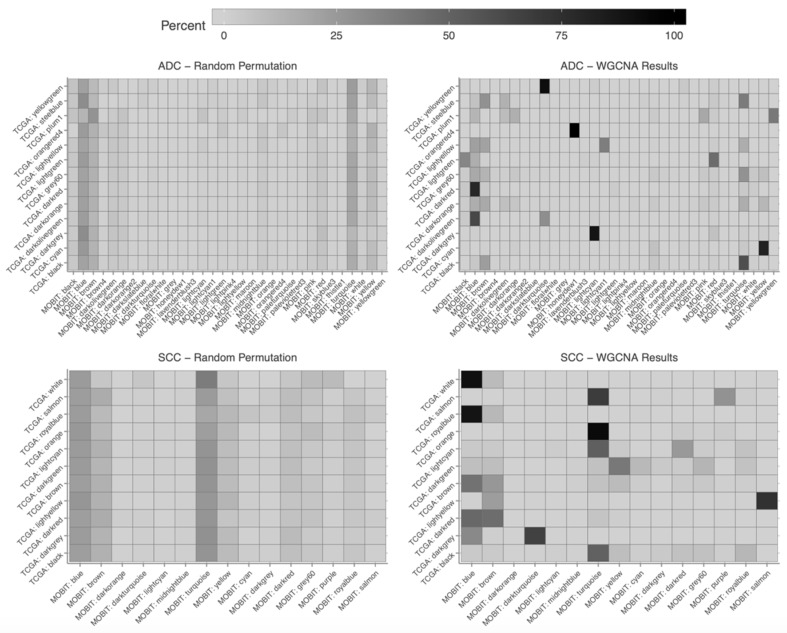
The percentage of genes allocated to a cluster based on TCGA (The Cancer Genome Atlas) data (rows) found in cluster build on MOBIT data (columns).

The co-expression network was validated on LUAC (lung adenocarcinoma) and LUSC (lung squamous cell carcinoma) subset from the TCGA database. No outlier was identified, and current soft-threshold was validated. The network was therefore built with identical parameters (Figure S4). The gene intersection matrices showed associations between modules found in TCGA or MOBIT datasets. This was further evidenced by comparing to the results after random permutation of attributed gene clusters in TCGA, which exemplified the expected intersection matrix in the absence of association between TCGA- and MOBIT-based networks. Finally, some modules obtained in TCGA could be strongly associated with corresponding gene module in MOBIT, thus validating MOBIT modules (e.g., TCGA yellowgreen to MOBIT darkturquoise for ADC, or TCGA white to MOBIT blue in SCC) (Figure 8).

### 2.6. Association of Modules with Clinical Traits

**Figure 9:**
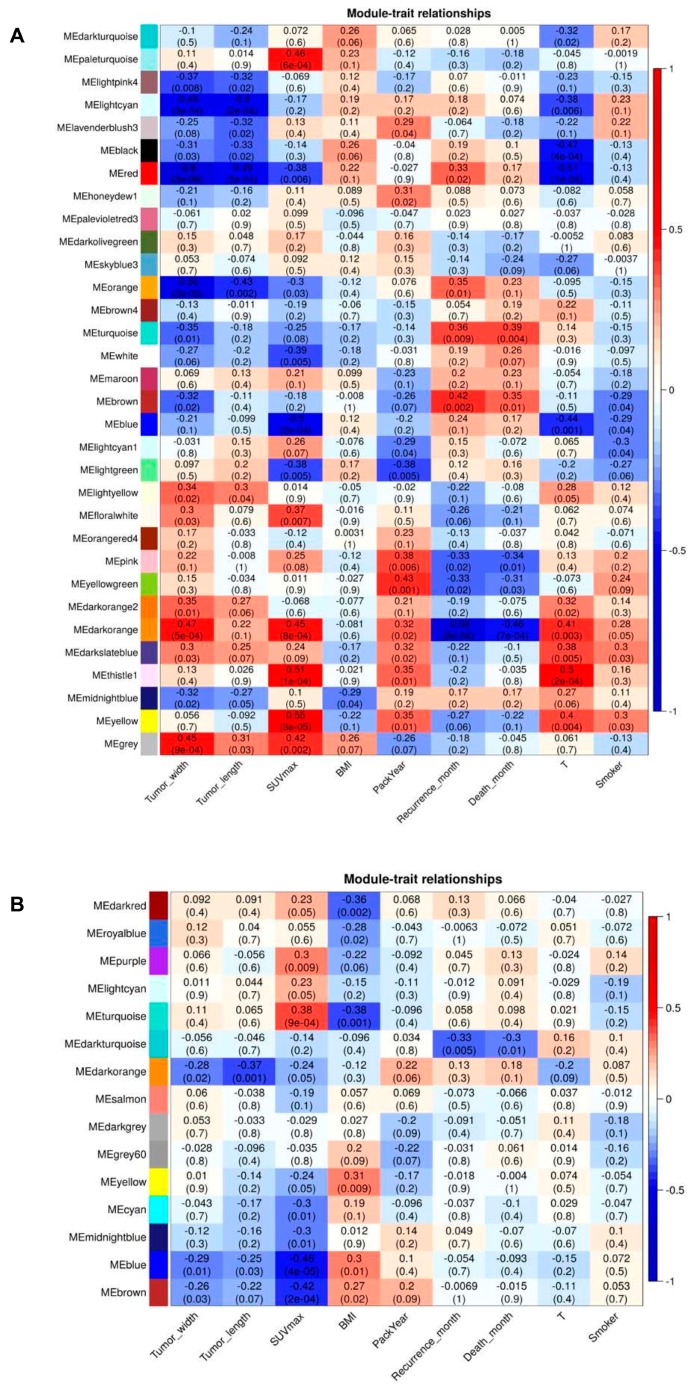
Heatmaps of the correlation between Eigengene and clinical traits of (**A**) SCC and (**B**) ADC. Each row corresponds to a module eigengene, and each column corresponds to a clinical characteristic. Each cell contains the corresponding correlation and *p*-value.

For each module, the gene co-expression was summarised by the Eigengene (i.e., the first component expression of genes belonging to that module) and we calculated the correlations of each Eigengene with clinical traits, such as tumour width, tumour length, SUV_max_, BMI (body mass index), pack-year, recurrence-free survival, disease-free survival, T, and smoking status. We noticed that there were four and two key modules most associated with one or more traits in ADC and SCC, respectively. As presented in Figure 9A, the red and lightcyan modules were strongly correlated with the tumour size in ADC, whereas the darkorange module had the highest negative correlation with the recurrence-free survival (RFS) in ADC. Furthermore, the yellow module, as well as the blue and turquoise modules had the highest correlation with SUV_max_ in ADC and SCC, respectively (Figure 9A,B). These results allowed selection of modules which were interesting for further analysis.

### 2.7. Functional Enrichment Analysis of Critical Modules

**Figure 10:**
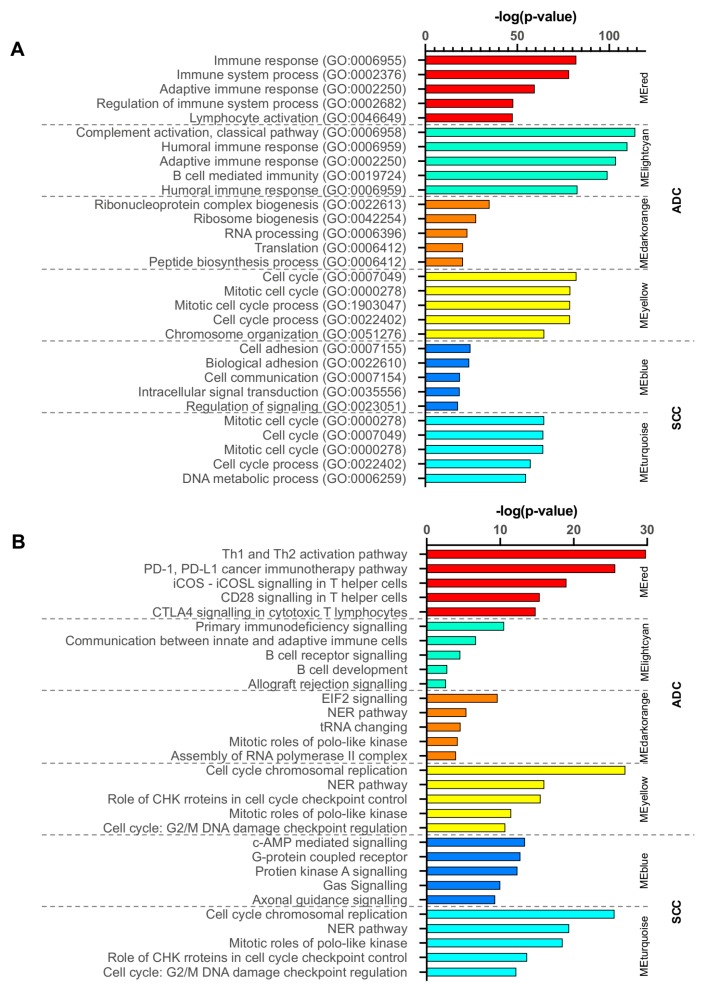
Top five enrichment results of (**A**) GO-Biological Process analysis by BINGO and (**B**) canonical pathway analysis by IPA for red, lightcyan, darkorange, and yellow modules in ADC, as well as for blue and turquoise modules in SCC.

The GO-biological process (BP) and canonical pathway analysis by IPA were performed in four modules (red, lightcyan, darkorange, and yellow) in ADC and two modules (blue and turquoise) in SCC. As shown in Figure 10A, the GO analysis results revealed that in ADC, red and lightcyan modules’ genes in the BP group were mainly enriched in immune response and the regulation of this process. For the darkorange module, which had strong negative correlation with RFS, the genes were mainly enriched in RNA biogenesis and processing. The mitotic cell cycle and DNA conformation change processes were enriched in modules yellow and turquoise in ADC and SCC, respectively. The blue module genes in SCC in BP group were mainly enriched in cell adhesion, communication, and signalling processes.

Following analysis by IPA revealed that in the red and lightcyan modules of ADC, canonical pathways were mainly related to the checkpoint of immune cell activation. The pathways in the darkorange module included the canonical pathways connected with the translation process. The enriched pathways in the yellow module in ADC, as well as in turquoise module in SCC were involved in cell cycle regulation process, involving cyclins and kinases. In SCC, the blue module included the canonical pathways connected with the signalling through G protein-coupled receptors and axon guidance molecules (Figure 10B).

### 2.8. Hub Gene Identification in the Selected Module

**Figure 11:**
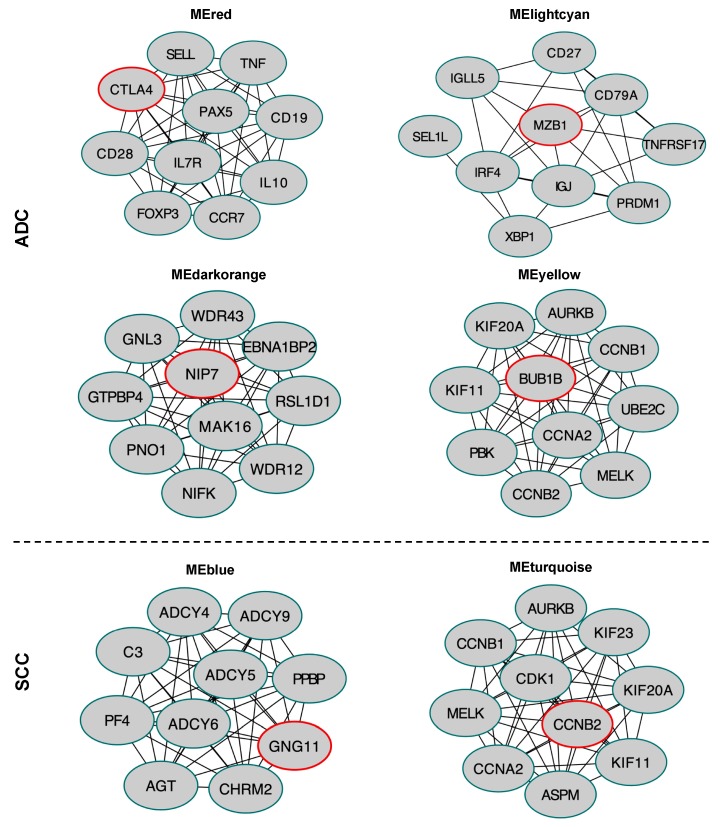
The networks of the top 10 hub genes in modules. Nodes represent genes and red colour indicates the highest rank.

In addition, we also constructed a protein-protein interactions (PPI) network of the expressed genes in the four selected modules in ADC (red, lightcyan, darkorange, yellow), in two selected modules in SCC (blue, turquoise) (Figures S5 and S6), and identified top 10 hub genes (Figure 11). The PPI network was generated using the STRING database in Cytoscape version 3.7.2 (http://cytoscape.org/), and 11 scoring methods including the newly developed algorithm Maximal Clique Centrality (MCC) were employed by use of *cytoHubba* plugin [42]. Hub genes with high betweenness centrality are important because they have the shortest path connectors through a network.

The top 10 hub genes in the red module, in ADC comprised *CTLA4, PAX5*, *CCR7*, *SELL*, *CD19*, *CD28*, *TNF*, *IL10*, *FOXP3*, and *IL7R*. For the red module, the highly connected hub gene was *CTLA4*, which is a member of the immunoglobulin superfamily and encodes a transmembrane protein which negatively regulates the T cell function [43]. In the lightcyan module, the top 10 high-degree genes were *MZB1*, *IGJ*, *CD79A*, *IRF4*, *IGLL5*, *PRDM1*, *XBP1*, *CD27*, *TNRFSF17*, and *SEL1L*. Most of the 10 hub genes in this module encode immunoregulatory factors. For instance, *MZB1*, with the highest degree of connectivity in the protein-protein interactions (PPI) network, promotes IgM assembly and secretion, as well as is a relevant component of cytoplasm involved in the regulation of apoptosis [44]. *NIP7*, *WDR12*, *EBNA1BP2*, *RSL1D1*, *GNL3*, *GTPB4*, *MAK16*, *NIFK*, *PNO1* and *WDR43* were identified in the darkorange module in ADC. *NIP7* encodes the nucleolar pre-rRNA processing protein which plays an important role in rRNA biosynthesis [45]. The top 10 hub genes in the yellow module of ADC included *BUB1B*, *CCNB2*, *NCAPG*, *KIF20A*, *KIF11*, *CCNB1*, *UBE2C*, *CCNA2*, *AURKB*, and *PBK*. *BUB1* encodes a serine/threonine-protein kinase that is a mitotic checkpoint protein [46].

We found the top 10 hub genes in the turquoise module in SCC including *CCNB2*, *KIF20A*, *KIF23*, *KIF11*, *CCNB1*, *CCNA2*, *AURKB*, *MELK, CDK1*, and *ASPM*. *CCNB2* encodes cyclin B2 protein, with the highest degree of connectivity in the PPI network in module turquoise in SCC, and may exert its effect by acting as a regulator of G2-M transition during the cell cycle [47,48]. In the blue module, the top 10 high-degree genes were *GNG11*, *ADYC9*, *ADYC6*, *ADYC5*, *ADYC4*, *C3*, *CHRM2*, *PPB2*, *PF4*, and *AGT*. The hub gene *GNG11* which encodes G protein subunit gamma 11 plays an important role in the transmembrane signalling system [49].

## 3. Discussion

In order to find the unique genes signature in each subtype of lung cancer, we did RNA-seq analysis in ADC and SCC samples compared with normal lung tissue in a well-defined cohort of NSCLC patients. Gene ontology (GO), canonical pathway analysis with the prediction of upstream regulators, and weighted co-expression gene network analysis (WGCNA) with the selection of hub genes were used to explain the most likely significant transcriptome profile alterations and to link them with the clinical traits. 

We identified 7720 and 5856 differentially expressed genes in lung ADC and SCC, respectively. The majority of genes were downregulated. Compared with lung adenocarcinoma, there were more DEGs in the lung squamous carcinoma tissues. The top upregulated genes in both subtypes of NSCL play a major part in cancer progression, as evidenced by human cancer studies. Among the DEGs identified in both subtypes, *MAGEA9* showed the greatest upregulation in tumour tissues. Moreover, it was also noticed that other genes from MAGEA family members were also significantly higher than normal tissues in both subtypes of NSCLC (*MAGEA1, MAGEA10, MAGEA11, MAGEA4*). Many studies have shown that melanoma-associated antigen (MAGE) genes, which are the best-characterised members of cancer/testis (CT) antigens family, have no expression in somatic adult tissues, but they differentially expressed in a variety of human cancers [50]. *MAGEA9* is frequently overexpressed in bladder cancer, cutaneous T-cell lymphomas, oesophageal adenocarcinomas, as well as in renal cell carcinoma [51,52,53,54]. Currently, there are only a few reports about *MAGEA9* in NSCLC. Zhang et al [55] showed that the high expression of *MAGEA9* protein in NSCLC tumour cells was present in SCC and it was correlated with tumour size and lymph node metastasis. Our results indicate that the members of MAGEA gene family, homologous *MAGEA9* in particular, may play key role in lung ADC and SCC development. If the results from further investigations from in vivo and in vitro studies support our findings, the combination of the appropriate strategy with targeting *MAGEA9* might be expected as a high efficacy of the chemo-immunotherapeutic approach. On the other hand, the lowest expression in SCC and ADC tissues was of *SFTPC* gene. Recent experiments have established that the expression of *SFTPC* was downregulated in human lung adenocarcinoma tissues and it was correlated with poor overall survival of ADC patients [56]. Overexpression of *CFBF*, *PAGE2*, *CALM3*, *DSG3*, *CTAG2*, and *FAM83C* was seen only in squamous cell carcinoma. The overexpression of *PAGE2* and *DSG3* was previously described in NSCLC. Djureinovic et al [57] confirmed the overexpression of PAGE2 protein in NSCLC tissues. Moreover, results from microarray-based studies have led to the identification of upregulated expression levels of the *DSG3* gene in squamous cell carcinoma.

To further identify the differentiating molecular mechanism of ADC and SCC, we focused on the biological pathways. The genes in both subtypes were mainly involved in biological processes associated with cell division and cell proliferation. However, IPA results have suggested that eicosanoid signalling, as well as agranulocyte and granulocyte adhesion and diapedesis signalling pathways, may be involved in the development of NSCLC. The top activated canonical pathway in lung ADC and SCC was eicosanoid signalling pathway (Figure S1). In general, eicosanoids are biologically active lipids which play key role in the various pathological processes, such as inflammation and cancer. For instance, prostaglandin E2 (PGE2) affects the development of cancer, primarily by suppression of immune response which leads to the induction of cellular proliferation. Interestingly, according to the previous study, there were significant differences in the extent of upregulation of mPGES-1 and its enzymatic product PGE_2_ was found to reveal lung tumours [58]. Agranulocyte and granulocyte adhesion and diapedesis, which is another pathway identified in the IPA, appears to be involved in the tumour immunosurveillance (Figures S2 and S3).

One of the mechanisms of avoiding immune surveillance by tumour is the suppression of the activity of adhesion molecules in the multistep adhesion cascade during the emigration of leukocytes from the bloodstream into the tissue [59]. In our study, we found that a variety of genes attending in the adhesion cascade (*CD34*, *E-selectin*, P*-selectin, ICAM1*, *ICAM2*, *JAM2*, *PNAd*, *chemokine*) were downregulated in lung SCC and ADC. The previous study evaluated the low expression of P-selectin protein correlated with diminished leukocyte tumour infiltration and poor prognosis [60]. Similarly, it was indicated that *ICAM-1* and *ICAM-2* suppression was positively correlated with decreased leukocyte extravasation and associated with cancer development [61]. In addition, a previous study with renal cell carcinoma by Hellwig et al [62] revealed that the expression of *CD34* was downregulated compared to normal renal tissue. Thus, our results indicated that NSCLC is significantly associated with mechanisms to escape immunity by tumour cells.

Many of the lung SCC-associated altered biological processes were involved in keratinisation, epidermal cell and keratinocyte differentiation. SCC is a malignant epithelial tumour, arising in tissues that provide a barrier between the organism and its environment [63]. At an earlier stage, lung SCC is accompanied by epidermal keratinisation. Park et al [64] have provided ample evidence that keratinisation is a significantly poor prognosis factor in lung SCC. Tumour with keratinisation was also more frequently observed among patients with a history of smoking. In addition, we found in our study that the top upstream regulators in lung SCC were *KDM1A* and *TP63* genes. *KDM1A* is overexpressed in numerous epithelial cancers and promotes proliferation, migration and invasion. Egolf et al [65] reported that its downstream targets were involved in epidermal differentiation and cornification processes. Kong et al [66] indicated that treatment with *KDM1A* inhibitor (2-PCPA) reduced tumour NSCLC cell proliferation and migration. On the other hand, *TP63* has been shown as a key player in homeostasis and development of squamous epithelium. Moreover, numerous investigations confirmed the upregulation of expression of *ΔNp63α,* the predominant *TP63* isoform in squamous epithelium, in squamous cell cancers, including head and neck, skin, as well as the lung. Cancer-related target molecules of *TP63* identified by IPA (Table S1) were correlated with the apoptotic process, keratinisation, tissue development, and cell population proliferation. There is evidence that *TP63* can promote cell survival and proliferation, and is involved in cancer formation and progression [39].

The top activated GO-terms and canonical pathways in ADC detected a unique lung ADC landscape wherein deregulation of certain pathways involved targets associated specifically with tumour immune response. Our results indicated that the MIF regulation of innate immunity and eNOS signalling were the most significant changed pathways in ADC. *MIF* as a proinflammatory cytokine was determined to be upregulated in all stages of neoplasia in most types of cancers and metastatic conditions. It has been revealed that MIF directly links inflammation and initiation of tumorigenesis [67,68]. Interestingly, the coordinated *COX-MIF-p53* axis affects tumour suppression downregulation, as well as COX-2 and PGE2 upregulation, finally leading to enhanced tumour growth, proliferation, and progression [69]. This crosstalk can be seen from Figure S3. Nitric oxide (NO) is an important cell signalling molecule whose level was frequently elevated in many tumours, including that of the lung [70]. Collected evidence has demonstrated the tumour-promoting role of NO in various types of cancer, including of the lung, spanning from tumour initiation of cellular transformation to tumour progression, through the metastatic cascade and resistance to radio/chemotherapy. It was also further demonstrated that high levels of serum NO were associated with advanced-stage lung cancer and poor survival rate of patients. Among numerous upstream regulators in lung ADC, we identified those that were associated with tumorigenesis and inflammation (Table S2). One of them was *FGF2* which affected downstream molecules like *BIRC5*, *CDC25A*, *CXCL2*, *CXCL12*, *CDC25A*, *AXIN2*, *MMP1*, *MMP9*, *MMP13*, and *MIF*. These molecules are related to a progression in various types of cancer. For instance, in small cell lung cancer, FGF2 increased the expression of anti-apoptotic proteins and triggered chemoresistance [71]. The activation of FGF receptors can activate multiple signal transduction pathways including PI3K, MAPK, and STAT pathway in prostate cancer, thereby causing tumour progression [72]. In addition, IL1B pro-inflammatory cytokine turned out to be a promising upstream regulator in lung ADC. It encodes the IL-1B protein which affects multiple aspects of the tumour microenvironment. IL-1B generated in the course of chronic inflammation supports tumour growth and metastasis predominantly by tumour-infiltrating macrophages [41,73].

Despite the fact that the traditional DEGs analysis has provided enormously relevant information, only the application of weighted co-expression network analysis (WGCNA) allowed for identifying the pattern of correlation among genes. WGCNA is notably useful for the identification of the modules of co-expressed genes that are correlated with clinical traits and consequently biological tumour behaviour. We identified four modules (red, lightcyan, darkorange, yellow) in ADC, and 2 modules (blue, turquoise) in SCC, the most related with tumour size (width, length), SUV_max_ parameter and recurrence-free survival (RFS). We found strong correlations of module red and lightcyan in ADC with tumour size. In addition, the results showed that the darkorange module in ADC was significantly associated with RFS. These results demonstrated that key genes within the above modules may serve as potential markers of tumour progression. We determined that the genes in the red and lightcyan module were enriched in the biological pathways involved in the regulation of immune response, complement activation, and altered T cells and B cells signalling. These key signalling pathways are closely related to the tumour microenvironment [74]. Similarly, Choi et al. [75] identified through WGCNA magenta and brown module of co-expressed genes in lung adenocarcinoma which were significantly correlated with tumour metabolism and immune microenvironment. The surge of interest in cancer immunotherapy is mainly focused on tumour immunosurveillance. Numerous studies have noted the tumour-infiltration of immune cells in the lung cancer microenvironment, but these cells play a dualistic role, they suppress and/or promote tumour progression and growth based on the production of growth factors, cytokines and chemokines into the tumour microenvironment [76].

To identify key genes in the five different colour modules, a PPI network was constructed and hub genes were identified with Cytoscape software and *cytoHubba* plugin, respectively. We found that *MZB1* was the top high-degree gene in ADC lightcyan module. According to previous studies, *MZB1*, an immunoregulatory molecule, was expressed at significantly decreased levels in gastric cancer (GC) tissues. It has been reported that siRNA-mediated knockdown of *MZB1* significantly increased proliferation, invasion and migration of GC cell lines [77]. The WGCNA used by Zhai et al [78] also demonstrated that *MZB1* was the hub gene in the module strongly associated with immune response in lung adenocarcinoma. According to the network analysis of the red module, *CTLA4* and *PAX5* were identified as the highest degree hub genes. Until recently, CTLA-4 was known as the inhibitor of anti-tumour immunity, but the effectiveness of the anti-CTLA-4 therapies is limited to a small number of cancer types [79]. However, it has been recently shown that *CTLA4* may play the tumour suppressor role across different cancer, including lung adenocarcinoma [80]. The protein encoded by *PAX5* acts as a nuclear transcription factor that is involved in B cell development. However, it is shown recently that *PAX5* was down-regulated significantly in lung adenocarcinoma but not squamous carcinoma [81,82]. Zhao et al [83] revealed that *PAX5* functions as a tumour suppressor in NSCLC cells, both in in vitro and in vivo assay. Interestingly, it was shown that re-expression of *PAX5* in lung cells inhibited β-catenin signalling pathway, which plays important role not only in proliferation, differentiation, and migration but also in regulating immune cell infiltration of the tumour microenvironment [84]. For the darkorange module of ADC, the most correlated with recurrence-free survival, *NIP7* was the highest degree gene. Interestingly, *NIP7* has been also identified as the gene among in a three-gene signature significantly associated with RFS in liposarcoma [85].

Next, we observed that the yellow module of ADC and the turquoise module of SCC were enriched in mitotic cell cycle and nuclear division biological processes and positively correlated with SUV_max_. SUV_max_ is the most common semi-quantitative parameter determined by ^18^F-FDG PET/MRI. SUV_max_ of a tumour is a product of several fundamental factors including glucose metabolism and the type or a number of cells present in the tumour. Kosal et al [86] reported that SUV_max_ of NSCLC tumour was positively correlated with the number of mitosis, the largest tumour diameter, and pathological stage of the disease. In consistence with those studies, the present study found that SUV_max_ was also positively correlated with genes involved in mitosis and cell cycle regulation process. *BUB1* was the highest connective gene in the PPI network in the yellow module of ADC, whereas *CCNB2* in the turquoise module of SCC. Both are mitotic genes activated during mitosis. CCNB2 which encodes the cyclin B2 protein is additionally involved in checkpoint control and its overexpression was positively associated with the tumour size and lymph and node metastasis in NSCLC [47,87]. The presence of top 10 genes from the yellow module of ADC and the turquoise module of SCC has been also identified as hub genes in NSCLC by Li et al [88] and Xu et al [89] investigations. Li and co-workers determined through WGCNA the survival-related module containing genes enriched in the cell cycle. In addition, these modules were defined as hub genes such as *MELK* and *UBE2C*, which were also identified in the yellow and turquoise module in our studies. Xu et al also observed *MELK, CCNB1, CCNA2, AURKA*, and *KIF11* as key genes associated with survival, which may be potential biomarkers or therapeutic targets for ADC. Furthermore, *AURKB*, *KIF23*, and *CCNA2* from the turquoise module of SCC were determined as hub genes by Gao et al through WGCNA in modules of lung squamous cell carcinoma that were strongly associated with cell division and overall survival [90].

In the blue module of SCC, which was also correlated with SUV_max_ parameter, the highest-degree gene was the *GNG11*. Low expression of *GNG11* was associated with worse overall survival for the female lung cancer patients who never smoked [91,92]. However, the specific biological function of *GNG11* in lung cancer still remains unknown.

## 4. Materials and Methods

### 4.1. Study Cohort

For this study, 114 patients underwent tumour resection surgery, during which two to three tissue fragments were collected. Pathological specimens were reviewed to confirm diagnosis of ADC, SCC or non-malignant lung tissue. Other inclusion criteria consisted of the material containing at least 50% of tumour cells for the RNA extraction; completely resected tumour (free resection margins); and follow-up including monitoring events for cancer recurrence, and lung cancer-related deaths. All patients underwent a PET/MRI examination. All study participants provided written informed consent and received detailed information on the study and associated risk prior to the enrollment. The study was conducted in accordance with the Declaration of Helsinki as a statement of ethical principles for medical research involving human subject and was approved by the local ethics committee of the Medical University of Bialystok, Poland (approval number: R-I-002/36/2014).

### 4.2. RNA Sample Preparation and Sequencing

Total RNA was purified from 114 tumour and 114 normal tissues with the RNeasy Mini Kit (Qiagen, Germany) according to the manufacturer’s instruction. RNA concentration, purity, and integrity were assessed by Qubit (Invitrogen, Carlsbad, CA, USA) and Tape Station 2200 (Agilent Technologies, Santa Clara, CA, USA). RNA-seq libraries were constructed from 1 μg of total RNA with RNA integrity number (RIN) >8, and using the Illumina TruSeq^®^ Stranded Total RNA Library Prep Gold (#20020598, Illumina, San Diego, CA, USA). Indexed libraries were pooled, clustered with the use of the cBot, and sequenced on the Illumina HiSeq 4000 platform generating 150 bp paired-end reads (2 × 75 bp). The quality of the obtained reads was assessed using FastQC (Babraham Institute, Cambridge, United Kingdom) before the analysis, as well as after different processing steps. As all reads had high quality, they were only soft trimmed and filtered with BBduk (US Department of Energy Joint Genome Institute, Walnuk Creek, CA, USA). BBduk tool was also used to filter out fragments originated from rRNAs. Cleaned reads were mapped to the human reference genome GRCh37 using splice-aware aligner STAR [93]. Counts per gene were estimated with RNA-Seq by Expectation Maximization version 1.3 [94] and used for data visualisation and further differential gene expression analysis. The quality of mapping was assessed with Qualimap version 2.2.1 [95], flagstat module from Samtools [96], and RSeQC [97]. Mapped reads were also visually inspected with Integrative Genomics Viewer [98]. Samples size factors were estimated, and counts were normalised using Relative Log Expression (RLE). Differential expression analysis assuming negative binominal distribution was performed with R packages DESeq2 [19], while the package EdgeR [23] (Trimmed Mean of M-values normalisation) was also used for verification of estimation robustness. After model fitting, dispersion estimates were obtained, and general linearised model was applied. Likehood ratio test (LTR) was used to call differentially expressed (DEGs) under |log2FC| ≥ 1 and false discovery rate (FDR) corrected *p*-value ≤ 0.05. Volcano plots generated were based on DESeq2 estimation and drawn using the R package ggplot2 v.3.2.1 (Hadley Wickham, RStudio Inc., Boston, MA, USA).

### 4.3. Functional Enrichment Analysis and Identification of Upstream Regulators

To understand the functional significance of dysregulated genes, the gene ontology (GO) enrichment analysis-biological processes (BP) by BiNGO tool [32] as plugin for Cytoscape version 3.7.2 (http://cytoscape.org/), the Ingenuity Pathway Analysis (IPA) (QIAGEN Inc., https://qiagenbioinformatics.com/products/ingenuity-pathway-analysis), and Metascape tool (http://metascape.org) [33] were used. BiNGO plug-in determined overrepresentative GO categories in a set of genes, based on a two-sided hypergeometric test with a Benjamini-Hochberg adjustment. IPA was used to perform the core analysis to identify canonical pathways and to predict the upstream regulators. Metascape is a web-based portal that provides a comprehensive gene list annotation based on 40 independent databases for studying functional enrichment. Venn diagrams and heat maps were created with the BioVinci Software (BioTuring, San Diego, CA, USA).

### 4.4. Construction of Weighted Gene Co-Expression Networks and Identification of Modules Associated with Clinical Traits

Gene co-expression network analysis was specifically performed on tumour tissues using the R package WGCNA [11,12]. The expression matrix was restricted to only expressed genes, when the number of reads sequenced was higher than 10 in a minimum of 15 samples, normalised for sample depth (count per million read, CPM) and log-transformed, including a pseudo-count of four (Log_10_(CPM + 4)). Then, the optimal soft threshold for adjacency computation was graphically determined. The transformed expression matrix was inputted into the WGCNA package functions, modules, and corresponding Eigengenes were obtained. The cutreeDynamic function was used for tree pruning of the gene hierarchical clustering dendrograms resulting in co-expression modules; correlated modules (r > 0.75) were then merged. The dissimilarity of the module Eigengenes (ME) was calculated using the moduleEigengenes function in the R WGCNA package. Association between Eigengenes values with clinical traits was assessed by Pearson’s correlation.

### 4.5. Protein-Protein Interaction Network Construction for Selected Modules and Hub Genes Identification

In order to identify highly connected hub genes in the protein-protein network (PPI), the *cytoHubba* plugin based on Cytoscape was used. At first, the correlation of the gene to a module Eigengenes, which is known as the module membership (MM), was assessed by Pearson’s correlation. In this study, hub genes were selected for MM > 0.55 in the specific module. 

## 5. Conclusions

Taken together, our RNA-seq data has generated a wealth of information for potentially new biomarkers, networks, and pathways dysregulated in lung ADC and SCC tissues which can be further explored to unravel the molecular mechanisms regulating the development of each subtype of NSCLC. Key biological processes, canonical pathways, and upstream regulators, which all play important roles in cancer progression, were predicted by bioinformatics tools. In addition, we applied WGCNA for exploring molecular networks associated with clinical traits such as tumour size, SUVmax, BMI, smoking status, recurrence-free survival, and disease-free survival. Overall, the results of this study emphasise the need to further explore the selected genes and pathways, particularly those related to the tumour microenvironment and immune escape mechanisms in ADC and SCC.

## Figures and Tables

**Table 1:** Patient baseline characteristics.

Characteristic	NSCLC patients (*n* = 114)
Age, year (mean ± SD)	65.4 ± 7.9
Male, *n* (%)	80 (70.2)
Smoking, PY (mean ± SD)	39.6 ± 21.0
BMI (mean ± SD)	26.9 ± 4.7
SUV (max) (mean ± SD)	8.8 ± 4.6
Histology	
Adenocarcinoma (AC), *n* (%)	45 (39.5)
Squamous Cell Carcinoma (SCC), *n* (%)	69 (60.5)
TNM stage, *n* (%)	
I	39 (34.2)
II	52 (45.6)
III	17 (14.9)
IV	6 (5.3)
Recurrence (%)	15.2
Deaths (%)	13.3

SD, standard deviation; PY, pack year; TNM, tumour node metastasis; BMI, body mass index; SUV, standardized uptake value; NSCLC, non-small cell lung cancer

## Supplementary Materials

The following are available online at https://www.mdpi.com/2072-6694/12/1/37/s1, Figure S1. Eicosanoind signalling pathway in lung SCC and ADC identified by IPA, Figure S2. Agranulocyte adhesion and diapedesis pathway in lung SCC and ADC identified by IPA, Figure S3. MIF regulation of innate immunity pathway in lung ADC identified by IPA, Figure S4. Cluster dendograms of the gene clusters of (A) LUAD and (B) LUSC subset from TCGA database, Figure S5. Protein-protein interaction (PPI) networks of genes in the red (A), lightcyan (B), darkorange (C), and yellow (D) modules in lung ADC. The networks were constructed using Cytoscape v. 3.7.2. software, Figure S6. Protein-protein interaction (PPI) networks of genes in the blue (D) and turquoise (E) modules in lung SCC. The networks were constructed using Cytoscape v. 3.7.2. software, Table S1. Upstream regulator analysis of DEGs in lung SCC predicted by IPA, Table S2. Upstream regulator analysis of DEGs in lung ADC predicted by IPA.
