# Supplementary material for: Molecular Signature of Subtypes of Non-Small-Cell Lung Cancer by Large-Scale Transcriptional Profiling: Identification of Key Modules and Genes by Weighted Gene Co-Expression Network Analysis (WGCNA)
Source: Cancers (Basel). 2019 Dec 21;12(1):37. doi: 10.3390/cancers12010037 (PMC7017323; doi:10.3390/cancers12010037)
Supplement: Supplementary file 1 [file cancers-12-00037-s001.pdf]

Supplementary Materials: Molecular Signature of Subtypes of Non-Small Cell Lung Cancer by Large-Scale Transcriptional Profiling: Identification of Key Modules and Genes by Weighted Gene Co-Expression Network Analysis (WGCNA)

Magdalena Niemira, Francois Collin, Anna Szalkowska, Agnieszka Bielska, Karolina Chwialkowska, Joanna Reszec, Jacek Niklinski, Miroslaw Kwasniewski and Adam Kretowski

A

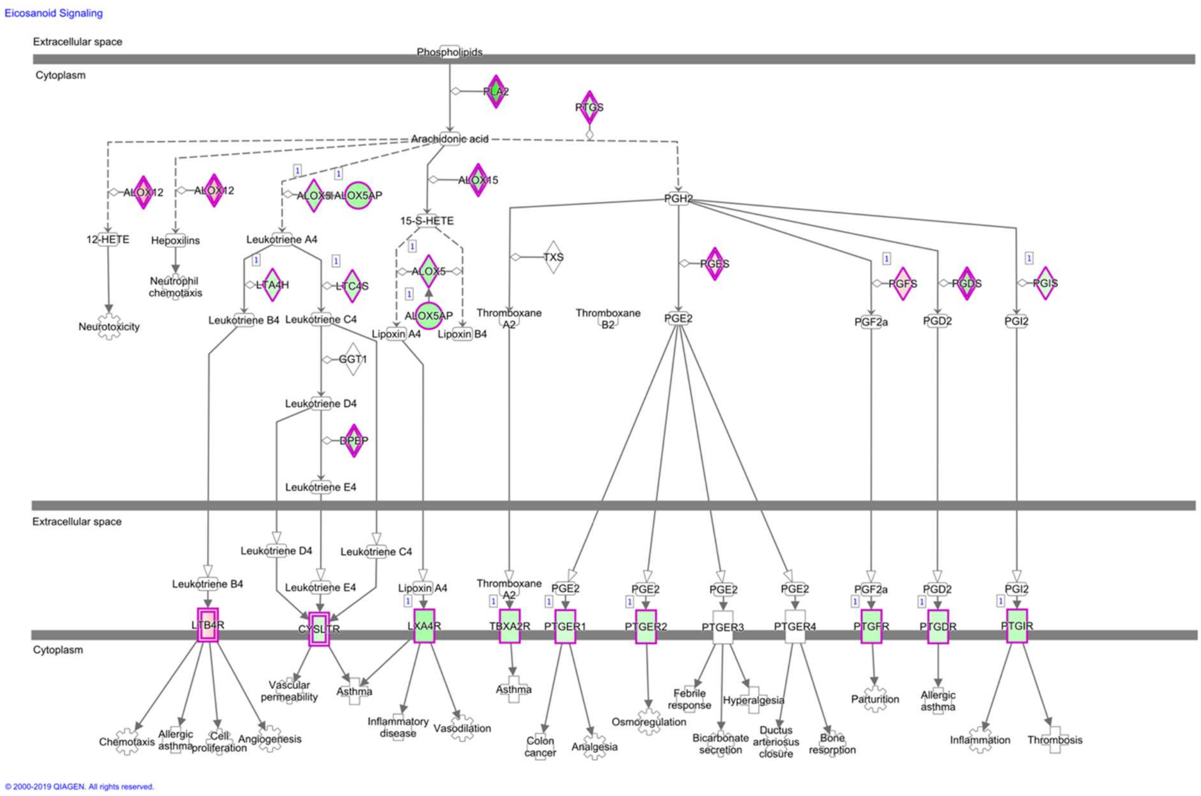

B

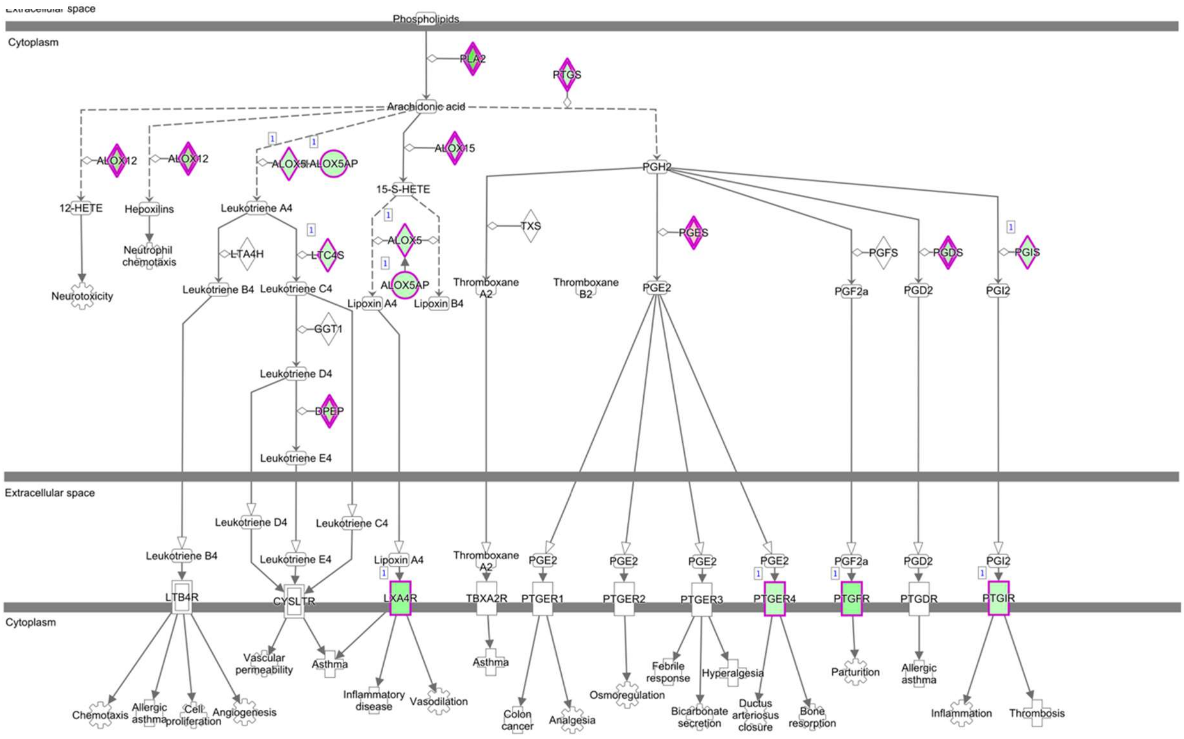

Figure S1. The top-ranked enriched canonical pathway identified in (**A**) SCC and (**B**) ADC using IPA: Eicosanoid signalling pathway.

Agranulocyte Adhesion and Diapedesis

A

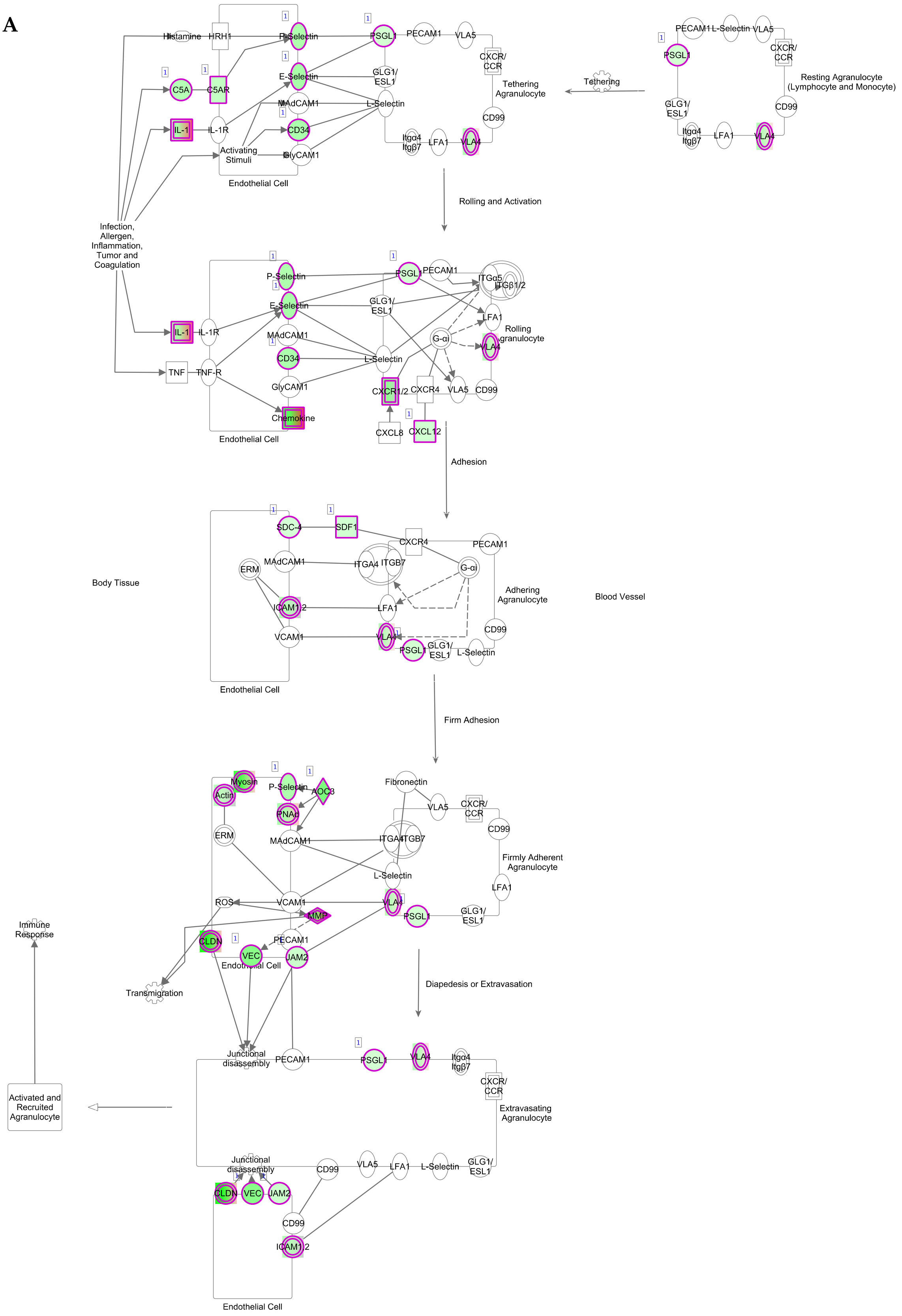

© 2000-2019 QIAGEN. All rights reserved.

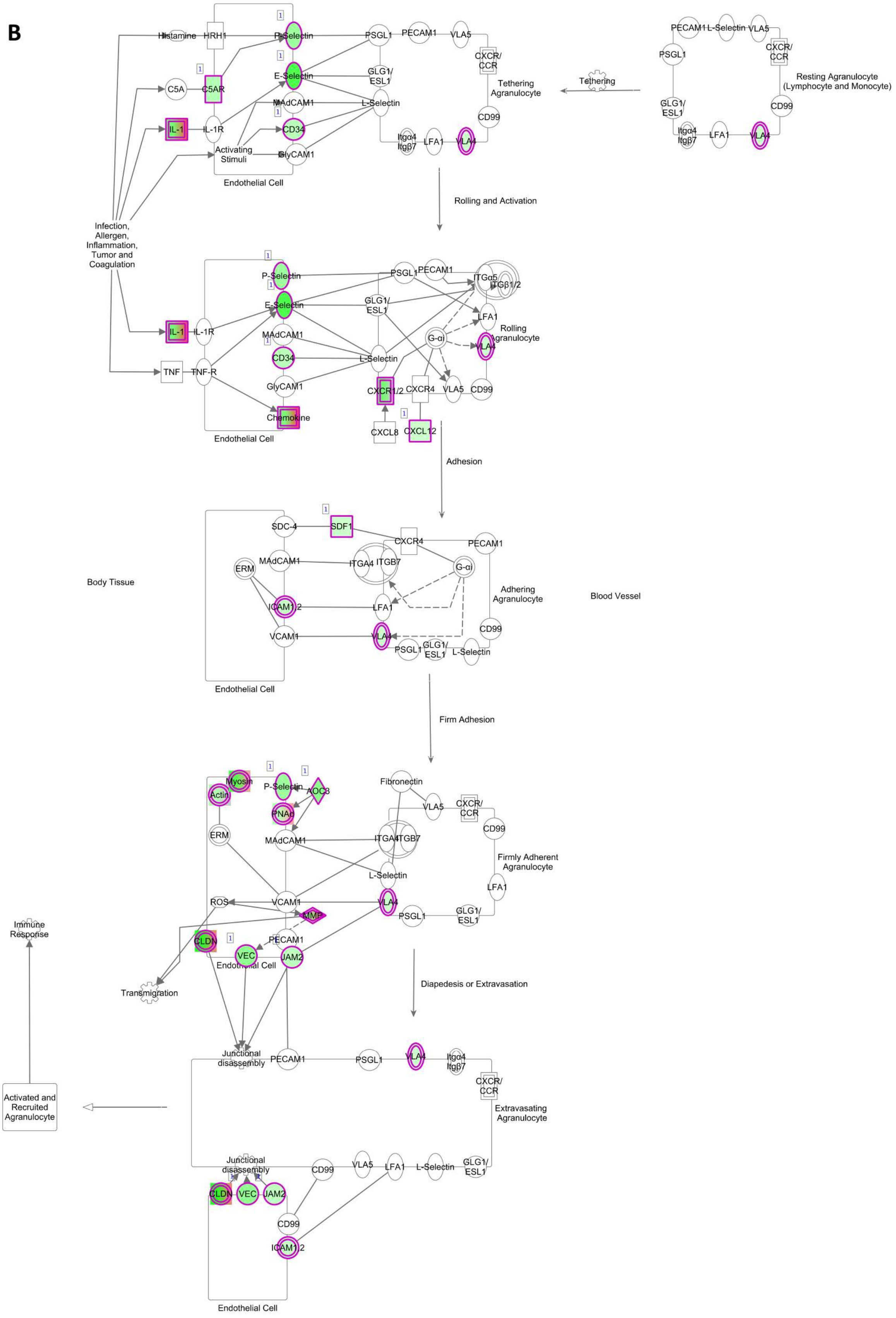

Figure S2. The second-ranked enriched canonical pathway identified in (A) SCC and (B) ADC using IPA: Agranulocyte adhesion and diapedesis.

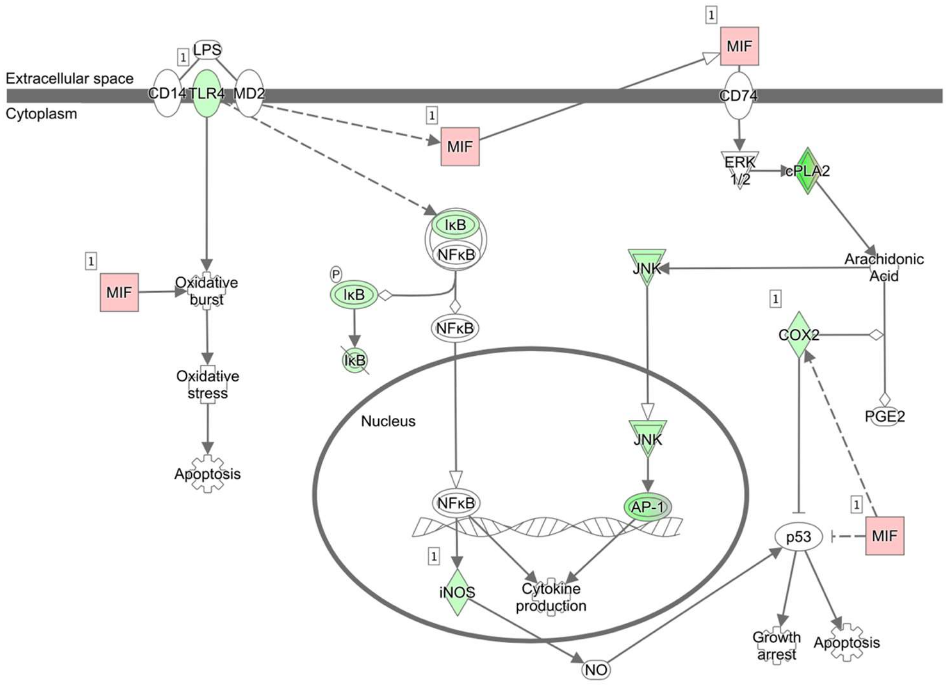

Figure S3. The top-ranked enriched canonical pathway identified only in lung ADC: MIF regulation of innate immunity.

A

B

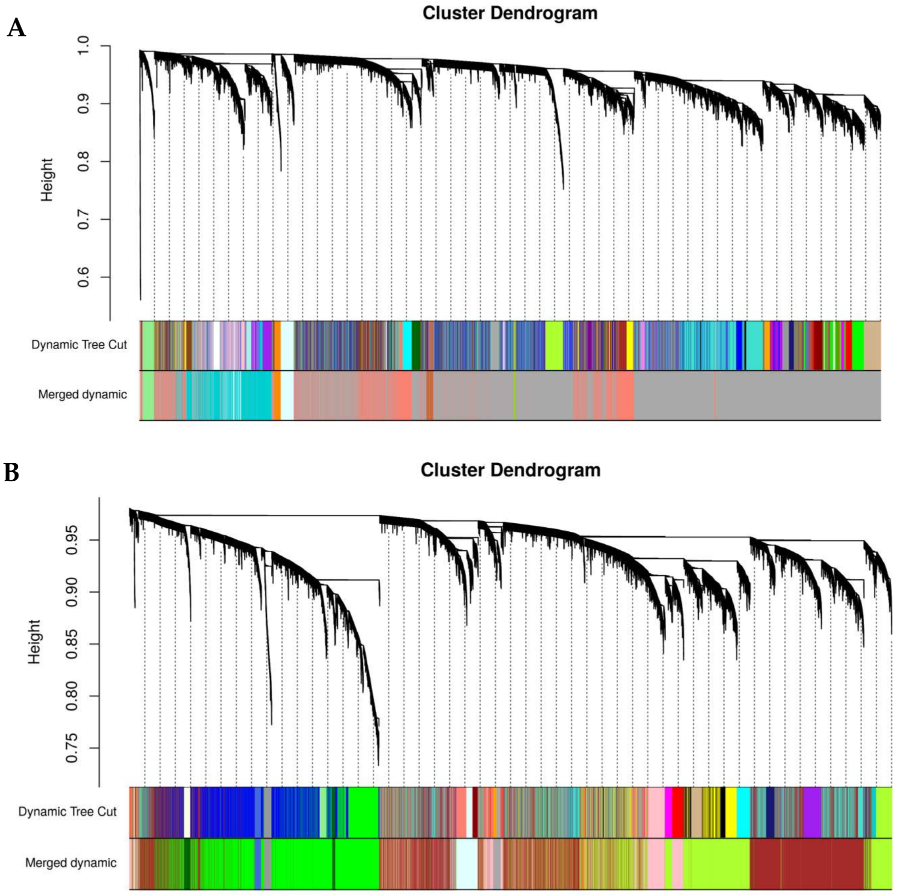

Figure S4. Cluster dendograms of the gene clusters of (A) LUAD and (B) LUSC subset from TCGA database.

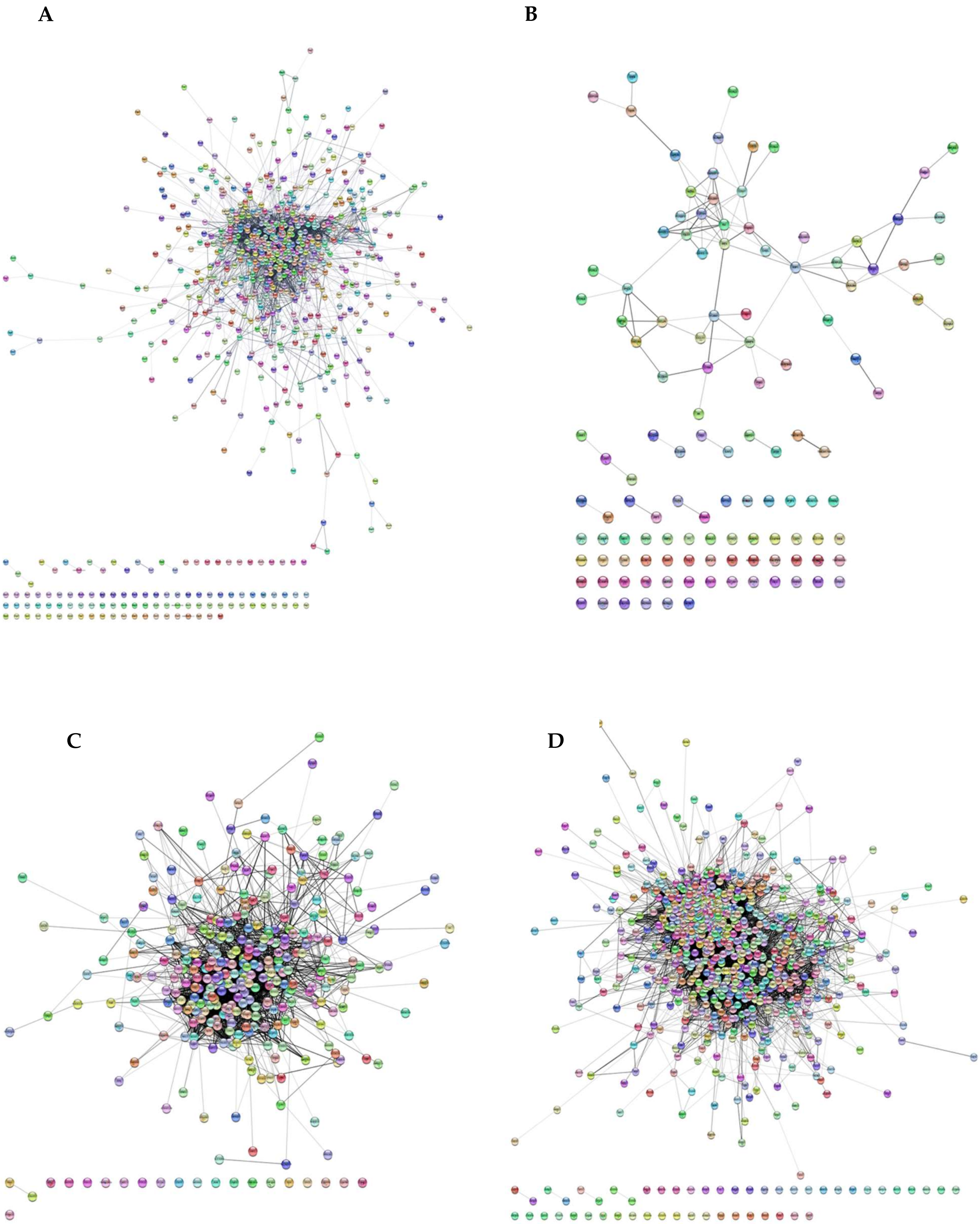

Figure S5. Protein-protein interaction (PPI) network of genes in the red **(A)**, lightcyan **(B)**, darkorange **(C),** yellow **(D)** modules in ADC. The networks were constructed using Cytoscape v. 3.7.2. software.

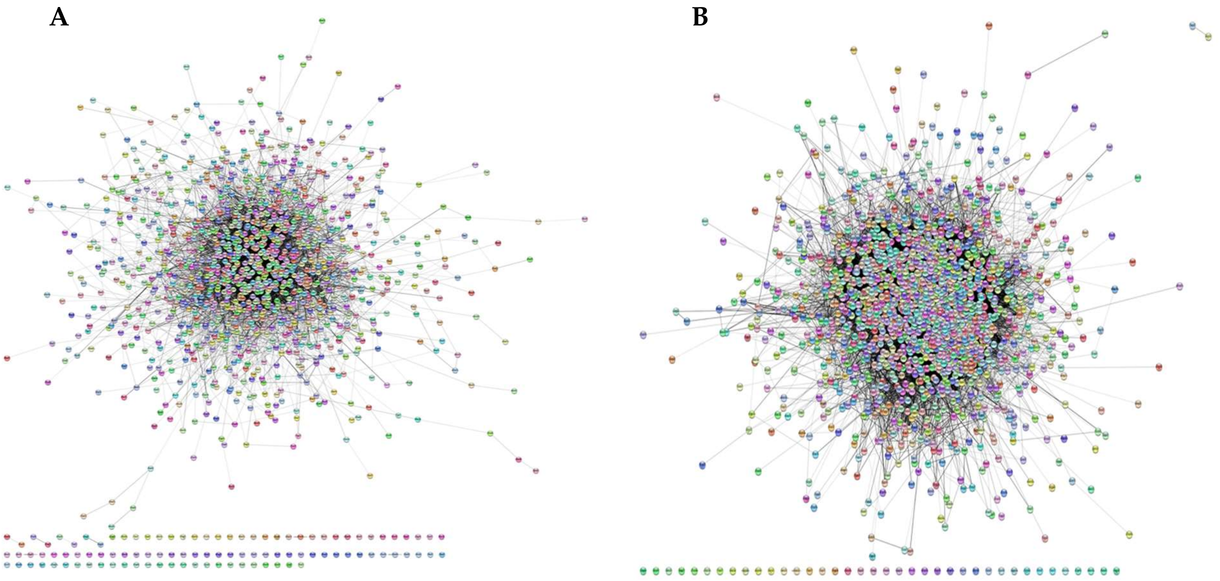

Figure S6. Protein-protein interaction (PPI) network of genes in the blue **(A)** and **(B)** modules in SCC. The networks were constructed using Cytoscape v. 3.7.2. software.

Table S1. Upstream regulator analysis of DEGs in lung SCC predicted by IPA.

Upstream Regulator	Prediction Activation State	Target Molecules in Dataset
**ERBB2**	Activated	ABCG2,ABRACL,ADAM12,ADAMTS16,ADRB3,AHNAK,AIMP2,ALDOC,ALOX15,ANG,AR,AREG,ASPM,ATP1B3,AURKA,BIRC5,BMP7,BNIP3,BRIP1,BUB1,BUB1B,C4BPB,CADM1,CAVIN1,CAVIN2,CCL2,CCN2,CCN5,CCNA2,CCNB1,CCNB2,CCND3,CCNE1,CCNE2,CD34,CD36,CD47,CDC20,CDC25A,CDC25C,CDC42EP2,CDC45,CDC6,CDC7,CDCA2,CDCA3,CDCA4,CDCA5,CDCA7,CDCA8,CDCP1,CDH3,CDK1,CDKN2A,CDKN3,CDT1,CENPA,CENPE,CENPF,CES1,CHDH,CHEK1,CHST2,CKS1B,CKS2,CLDN3,CLEC3B,CNTD1,COL1A1,COL3A1,COL5A1,COL5A2,COL7A1,CRIP1,CSE1L,CSF2,CXCL10,CXCL12,CXCL3,CYP4B1,DHH,DPEP1,DPT,DSG2,DUSP6,E2F1,E2F2,E2F3,E2F7,E2F8,EDN1,EDN2,EGR1,EIF4EBP1,EIF6,EMP3,EPHB2,ERBB4,EREG,ESPL1,ESR1,ETV1,ETV4,FEN1,FGF7,FGF9,FOLR1,FOS,FRRS1,FSCN1,FSTL3,FXYD3,FZD4,GART,GAS6,GBP6,GINS1,GINS2,GINS3,GINS4,GPC1,GPX3,HBEGF,HES2,HEYL,HIF1A,HIST1H4C,HMGA2,HP,HSD17B11,HSPB1,HSPB8,IGFBP3,IGFBP5,IL6,IL6R,IL6ST,IRF6,ITGA2,ITGA6,JAG1,JAG2,JAM2,JUN,JUP,KDM5B,KDR,KIT,KLF4,KRT7,KRT81,LAMC2,LDHA,LIG1,LPCAT1,LRRFIP1,LSR,LTBP2,MACF1,MAOA,MAPK4,MAST1,MCM10,MCM2,MCM3,MCM4,MCM5,MCM6,MCM7,MCM8,MFAP2,MFNG,MIF,MITF,MKI67,MME,MMP1,MMP10,MMP11,MMP13,MMP14,MMP3,MMP7,MMP9,MUC1,MUCL1,MYBL2,MYCN,MYL9,NCAPD2,NCAPG,NDC80,NDRG1,NDST1,NEDD9,NEK2,NFIB,NOTCH4,NOX4,NPNT,NPTX2,NRP1,ORC1,ORC6,PAICS,PCDHGA12,PCNA,PDIA4,PDK1,PDK4,PDLIM4,PENK,PFN2,PGK1,PLAC8,PLAU,POLA2,POLD1,POLD2,POLE2,POLQ,PPARG,PPAT,PRC1,PRDX2,PRIM1,PRIM2,PRR15L,PRSS3,PTGES,PTGS2,PTPRB,QKI,RAD51AP1,RARG,RECK,RFC2,RFC4,RFC5,RHOB,RHOD,RRAD,RRM1,RRM2,S100A4,SCG5,SDC1,SEC61A1,SHROOM3,SKP2,SLC1A6,SLC2A1,SLPI,SMAD7,SMC2,SNAI2,SOCS2,SORBS1,SOX4,SPAG4,SPAG5,SPARCL1,SPINT2,SPOCK1,SRPX,ST3GAL6,TERT,TFAP2C,TGFA,THBD,THSD1,THY1,TIMP3,TJP1,TK1,TNC,TNS4,TOP1MT,TOP2A,TOPBP1,TPD52,TPD52L1,TUBA1A,TUBB4A,TYMS,UBE2C,UGT1A6,VEGFD,VIM,VWF,WFS1,WNT5A,WNT5B,WT1,WWC1,ZEB1,ZNF703,ZWINT
**RABL6**	Activated	AURKB,BIK,BUB1,BUB1B,CCNA2,CCNB1,CCNE2,CDC25A,CDC25C,CENPF,CHEK1,CHEK2,CKS1B,DAPK1,DRAM1,EZH2,FEN1,FERMT2,H2AFX,HBEGF,HMMR,KIF23,MAD2L1,MCM10,MCM2,MCM5,MCM7,MELK,NCAPG,NDC80,NEK2,PBK,PLK1,PMAIP1,POLD1,POLE2,PRC1,PRIM1,PTPRM,RAD54B,SERPINH1,SMAD6,TOP2A,TPX2,TTK,UBE2C,VRK1
**CCND1**	Activated	AHNAK,AR,AREG,ARHGEF2,ASPM,ATAD2,ATP2B1AS1,AURKA,AZGP1,BIRC5,BRCA1,BRIP1,C7,CCNA1,CCNA2,CCND3,CCNE1,CCNE2,CDC45,CDC6,CDCA2,CDCA7,CDCA8,CDK4,CDKN2A,CENPF,CENPH,CENPK,CENPN,CEP55,CIP2A,CLSPN,COL1A1,COL5A2,CPED1,CST4,DDIAS,DEPDC1,DKK2,DONSON,DTL,E2F1,E2F7,E2F8,EIF5A2,ENO1,EPGN,EREG,ESCO2,FAM83D,FGF7,FOXM1,GNMT,GPRC5A,GPRIN2,HIST1H4H,HIST2H2BE,HJURP,HSPB1,HSPB8,ITIH2,JPT1,KDM6B,KIF11,KIF20A,KIF20B,KIF2C,KIF4A,KLHDC1,KNL1,KRT10,KRT14,KRT4,LAMB2,MACF1,MAFF,MAGI2,MARK1,MC4R,MCM10,MCM4,MCM7,MELK,MMP3,MTFR2,MYLK,MYO7A,MYRIP,NANOG,NCAPH,NOP2,PAH,PCLAF,PCNA,PDLIM3,PDLIM4,PGR,PLK1,PPARG,PRIM2,PRSS1,PSMC3IP,PSRC1,QKI,RAB3B,RACGAP1,RAD51,RBMS3,RFC5,RGS2,RMI2,ROR2,RRM2,SCG5,SELENBP1,SLC4A4,SOX2,SOX4,SPC25,SPP1,ST6GALNAC5,STOM,STXBP1,TAPT1,TGFBR2,THNSL1,TMEM204,TMPRSS2,TOR3A,TPI1,TPX2,TRAIP,TRIP13,TRPM8,TUBA1C,TUBB,TUBB1,TYMS,UTRN,WNT7A,ZNF367,ZNF423

**FOXM1**	Activated	ACE,AR,AURKB,AXIN2,BIRC5,BRCA2,BRIP1,BUB1B,CAT,CAV1,CCNA1,CCNA2,CCNB1,CCNB2,CCNE1,CCNE2,CCNF,CDC20,CDC25A,CDC25C,CDCA2,CDCA8,CDK1,CDKN2A,CDKN3,CENPA,CENPE,CENPF,CKS1B,CKS2,CXCL12,CYP7A1,ESR1,FOS,FOXF1,GTSE1,IL1RN,IL6,JUN,KDR,KIF20A,LAMA4,LDHA,LEF1,MCM8,MKI67,MMP7,MMP9,MUC5AC,MYCN,NEK2,NES,PCNA,PGK1,PLK1,PLK4,PRC1,PRDX2,PTTG1,RMI1,SFTPB,SFTPD,SKP2,SNAI2,SOX2,STMN1,TGFBR2,TJP1,TOP2A,TWIST1,VIM,ZEB1,ZEB2
**PTGER2**	Activated	ADGRE1,AREG,ASPM,AURKA,BRCA1,BUB1B,CCNA2,CCNB2,CDKN3,CENPE,CENPF,CEP55,CFP,CKAP2L,CKS2,CLEC4D,CXCR2,DEPDC1,ECT2,EGR1,FOXP3,FPR1,H2AFX,HAMP,HDC,HIST1H2AD,IL23A,IL36G,IL6,ITGAL,KIF11,KIF15,KIF18B,KIF20A,KIF22,KIF2C,MELK,MKI67,MMP9,NEK2,NUF2,NUSAP1,PBK,PGR,PLK1,PRC1,PTGES,PTGS2,RACGAP1,SPAG5,SPP1,STAR,STIL,TIMP1,TIMP3,TPX2,TREM1,TROAP,TTK
**SOX2**	Activated	ABCC6,ABCG2,ALOX5,ANGPT1,AQP4,ARHGEF10,ASCL2,ATF3,AXIN2,BFSP2,BIRC5,BMP4,BMPER,C1QL1,CASKIN2,CCN2,CCN4,CCN5,CDKN2A,CITED2,CLDN11,CNFN,COL1A1,COL9A3,CRABP2,CST6,CTNNAL1,CXCL14,CYP26A1,DDC,DKK1,DLX1,DLX2,DLX3,DLX4,DLX5,DNMT3B,DSP,DUSP1,EFEMP1,EFS,EN1,EPB41L2,ETV4,FABP7,FAM107B,FGF5,FGFR4,FOS,FOXA2,FOXD3,FOXE1,FOXF1,FOXH1,FST,GAB1,GAB2,GADD45B,GAL3ST1,GATA2,GATA6,GBP4,GBX2,GJC2,GPR17,GRHL3,GSC,HOPX,HOXC4,IER5L,IL23A,INAVA,IRF6,JAG1,JAM3,JUN,KDR,KIF26A,KLF4,KLF6,KRT14,KRT17,KRT19,KRT27,KRT6A,KRT7,LEF1,LEFTY1,LHX2,LHX5,LIMS2,LMCD1,MAG,MEF2C,MEIS1,MFSD2A,MIXL1,MSX2,MYCN,MYRF,NANOG,NEU4,NFASC,NIPAL4,NKX21,NKX23,NOTCH4,NR0B1,NR5A2,NRARP,NRP1,NTF4,OLIG1,ONECUT1,OTX1,OVOL1,PARD6B,PARD6G,PAX6,PCDH15,PDK1,PEG3,PIK3R1,PITX2,PLA2G16,PLAC1,PLD1,PLLP,PLP1,PLXNB3,POU4F1,PPARG,PSAT1,QKI,RAP1A,RASGEF1B,RNF43,S1PR5,SALL4,SAPCD2,SEMA5A,SEMA6A,SERPINA1,SNX22,SOX17,SOX2,SOX21,SPRR1A,SPRR3,SPRY4,STRA6,TBX3,TDGF1,TEC,TGFB2,TIMP3,TMEM100,TMEM163,TNFRSF19,TNNI1,TNR,TP63,TSPAN2,TUBB3,TWIST1,UGT8,VIM,WNT3,WNT5A,ZIC2
**KDM1A**	Activated	ACSM1,ADCY5,ADM,AGT,ARNTL2,ASF1B,ATAD2,AZGP1,BARX2,BLM,BMP2,BRCA1,BRCA2,BRIP1,C1orf116,CAD,CCNA2,CENPF,CENPK,CENPU,CHAF1B,CLCN2,COL1A1,COL1A2,CRYM,CSGALNACT1,DKK1,DNMT3B,E2F8,EBF1,FGF10,FOXA2,FOXM1,GATA5,GFI1B,GNMT,HAS3,HBB,HBG2,HOXB7,IRAK3,KLF2,LATS2,LEFTY1,MCM2,MCM3,MCM4,MCM6,MCM8,MSX2,MYBL2,NEBL,NEK3,NFE2L3,NOS3,ONECUT2,PRKG2,PSAT1,PTPRB,PTTG1,RARG,RCOR2,RETN,RHOU,RUVBL1,S100A7,SCN1A,SELENOP,SFRP2,SFRP4,SFRP5,SLC22A3,SMAD7,SOCS2,SOX2,SPOCK1,SUV39H1,SYBU,SYNPO,TCF19,TENM1,TERT,TIMELESS,TJP3,TOX2,TRIP13,ZBTB20,ZNF367
**TP63**	Activated	ABCB1,ACTL6A,ADA,ADAMTS1,ADH7,ADM,ALOX12,ANOS1,AREG,ARG1,BLM,BMP7,BRCA1,CA4,CAD,CCN1,CCNA2,CCNB1,CCND3,CCNE1,CDC25C,CDK1,CDKN1C,CDKN2A,CFLAR,CITED2,CKS2,CLDN3,COL4A3,COL5A1,CSTA,CXCL17,CYGB,CYP2A6,CYP2F1,DDIT4,DKK1,DLX1,DUSP6,E2F1,EFNA4,ENG,FANCA,FDXR,FGFR3,FOS,FOXA2,FST,G6PD,GAPDH,GCNT3,GPX2,GREM1,GRHL2,HAS3,HBEGF,HMGA2,HOXC4,IGFBP3,IHH,IL6,INSM1,ITGA2,ITGA7,ITGB4,ITGB8,IVL,JAG1,JAG2,KCNG1,KCNK1,KIF23,KLF6,KRT10,KRT14,KRT20,KRT31,KRT6A,KRT6B,KRT6C,KRT7,MAD2L1,MAFF,MCM10,MIR205HG,MLPH,MMP13,NANOG,PAK1,PAX9,PCNA,PERP,PI3,PKP2,PLAU,PMAIP1,POLD2,POSTN,PPBP,PRKCZ,PTHLH,RACGAP1,RAD51,RBBP8,RCC1,RGS13,RUNX2,S100A2,S100A4,S100A7,SERPINB2,SERPINB5,SFN,SLC7A11,SNAI2,SPATA18,SPRR3,STX1A,TERT,TGFB2,TGFBR2,THY1,TIMP3,TNC,TNS4,TP53AIP1,TP63,TP73,TRIM29,TWIST1,UGT1A1,UGT1A7 (includes others),ULBP2,UPK1B,VIM,WNT5A,ZEB1,ZEB2

**EP400**	Activated	CCNA2,CCNF,CDC20,CDC25A,CDC6,CDCA3,CDKN2A,CENPF,E2F1,E2F2,E2F7,E2F8,FABP4,FBXO5,FOXM1,H2AFZ,INCENP,MAPK12,MCM3,MCM4,MYBL2,NCAPG2,NEK2,PCNA,PLK1,PPARG,PSRC1,RCC1,SGO1,SKA1,SKP2,SUV39H2,TBX21
**RARA**	Activated	ACACB,ADH1C,ALDH1A2,ALOX5,APOA1,AR,ASPM,ATF5,BIRC5,BMP8A,C5,CA12,CALB1,CCN6,CCNA1,CCNA2,CD93,CDK1,CDKN3,CDX1,CEACAM6,CENPA,CENPF,CENPM,CENPN,CENPU,COBL,CP,CRABP2,CSF3,CTNNA3,CXCL5,CXCR5,CYP26A1,CYP7A1,DUSP1,E2F1,E2F7,E2F8,EGR1,EGR3,EIF5A2,EPHX4,F12,FGFR4,FOS,FOXD3,FOXP3,GAP43,GJB4,GPR158,HELLS,HIST1H4C,HLAJ,HOXA1,HOXA10,HOXA2,HOXA4,HOXA5,HOXB7,HOXB8,HOXD10,IL12RB2,IL13,IL16,IL5RA,IL6,IL6R,IRF6,JUN,JUP,KIF23,KIRREL2,KLF3AS1,KLF4,KNL1,KRT15,KRT20,LIFR,LMO3,LTF,LYPD1,MAD2L1,MALL,MAOB,METTL7A,MGP,MMP11,MND1,MYCN,NANOG,NCAPG,NCAPH,NEDD9,NR2F1,NRP1,ONECUT2,PBK,PCK1,PENK,PIMREG,PITX2,PLK4,PLXNA2,PTGS2,PTTG1,RAP1A,RARA,RARG,RASD1,RASGRP4,RBP1,RBP2,RORC,SALL4,SAMHD1,SCGB3A1,SFTPB,SHANK2,SLC7A11,SMC4,SOX17,SOX2,SPP1,STRA6,TERT,TFAP2A,TFF1,TG,THBD,TP63,UBE2T,UBL3,UBXN10,YPEL1,ZMAT4,ZNF367,ZNF703
**EHF**	Activated	ALOX5,ANPEP,BLNK,BMP4,CCBE1,CDH3,CDK1,CDKN2A,CNFN,COL2A1,CRABP2,CYP1A1,E2F1,EREG,FCER1A,FOLH1,FOXN1,GATA1,GATA2,GRHL3,HIST1H4H,HOPX,IL13,IL1RN,IL6,JAG1,KDR,KIT,KLK6,KLK8,MMP1,MMP3,MS4A2,MUC1,PGLYRP3,PGLYRP4,RHCG,S100A12,S100A7,S100A9,SAA1,SAA2,SCEL,SPRR1B,SPRR2A,SPRR2D,SPRR2E,SPRR3,TFPI,THBD,THY1,TIMP1,TNC
**HOXA10**	Activated	ADM,AIMP2,ALAS2,ALDH1A2,ALOX5AP,ALPL,ALPP,AQP1,ATP1A2,BCHE,CA3,CD1C,CDH16,CFD,CLDN5,COL3A1,CSF3R,CSTA,CTSG,CTSV,CYBB,CYP2F1,DKK1,DPP4,ERG,FST,FZD5,GAS6,GATA1,GSTA3,HBG1,HLF,HMBS,HOXA11,HOXA5,HOXD9,IDH2,IGFBP3,IGFBP5,IL11,INMT,KCNAB1,KLF9,KRT15,KRT4,LAMA2,LEPR,LTF,LYZ,MAPK12,ME1,MFNG,MIR99AHG,MYCN,MYLK,NCAM1,NCF2,NDRG2,NR4A1,P4HB,PCP4,PEG3,PHGDH,PIGR,PIK3R1,PROS1,RBP1,REM1,RNASE4,S100A12,SFRP4,SHD,SLC27A6,SNRPE,TDO2,TGFBR3,THBD,TLX1,TNXB,WNT10B,XDH
**GATA4**	Inhibited	ACTC1,ACTG2,ACTN2,AGTR1,ANKRD1,CACNA1C,CASQ2,CCBE1,CCK,CCL2,CCN2,CDCA8,CLDN1,CLDN2,CNFN,COL1A1,COL1A2,COL3A1,CRABP2,CTHRC1,CXCL2,CXCL6,CYP17A1,DAB2,DES,DIO2,DLX3,DSCC1,ECM2,EDN1,EPOR,ESPL1,F10,FAP,FGF2,FOS,FSTL4,GBX2,GRHL3,HSPB7,IL11,IL6,INHA,IRF6,JUN,KCNA5,KRT14,KRT17,KRT27,KRT7,LCT,LHCGR,LHX5,LRRFIP1,LTBP2,MEF2C,MSX2,MUC4,MYH6,MYL4,NFATC2,NKX25,NR5A2,NTF4,NUSAP1,OVOL1,PDLIM3,POSTN,PTGS2,PTPRB,RYR2,S100A4,SFTPC,SGPL1,SLC10A2,SLC8A1,SLC9A3,SPP1,SPRR1A,SPRR3,STAR,TAL1,TIMP1,TNNC1,TNNT2,TTN,VIM,ZIC2
**GATA6**	Inhibited	ABCB1,ACTC1,ALOX15,AMHR2,AQP5,ARG1,ASPA,BMP4,BMP5,BMPR2,CAV1,CD5L,CDCA8,CLEC10A,CNFN,COX5A,CRABP2,CXCL13,CYP17A1,DAB2,DLX3,DPP4,DSCC1,EDN1,ESPL1,FGF2,FOXA2,FOXD3,GATA6,GBX2,GPI,GRHL3,HBA1/HBA2,HYAL1,IRF6,KDM5B,KLF2,KRT14,KRT17,KRT27,KRT7,LAMA1,LAMB2,LEFTY1,LHCGR,LHX5,LRRFIP1,LYVE1,MARCO,MEF2C,MSX2,MYH11,MYH6,MYLK,NANOG,NAPSA,NFIA,NOS3,NR5A2,NTF4,NUSAP1,OVOL1,PGK1,PGM2,PPP1R14A,PTGES,SCGB1A1,SFTPA1,SFTPC,SHH,SLC9A3,SLPI,SOX17,SOX2,SOX7,SPRR1A,SPRR3,STAR,STARD13,TGFB2,TNNC1,VSIG4,WNT2,WNT7B,ZIC2
**SMARCA4**	Inhibited	A2M,ABCB1,ABLIM3,ABRACL,ACE,ACSL5,ADGRG1,AGMAT,AGT,AHNAK,AIM2,ALDH2,ANXA10,APOA1,APOL3,AREG,ARL4C,ASNS,AXIN2,AZGP1,BAK1,BIRC5,BMP4,CARD17,CCDC153,CCDC169,CCDC9B,CCL2,CCN2,CCNA2,CCNE2,CCR8,CCRL2,CD19,CD52,CD74,CDC25A,CDC6,

		CDH16,CDKN2A,CFP,CHEK2,CHODL,CKM,CLIC2,COL1A1,COL7A1,CP,CPM,CSF1,CTSH,CXCR5,CYP1A1,CYP26A1,CYP4B1,DCT,DES,DLX2,DUSP13,DUSP6,DYSF,E2F1,EBF1,ECM2,EDNRA,EGR1,EMP3,ENTPD3,EPHA1,EPHB2,EREG,ESPNL,EVA1A,FABP4,FAM167A,FBP1,FGF9,FGG,FHL2,FMO2,FOLR1,FOS,FOXA2,GADD45B,GAPDH,GCAT,GCLC,GJB5,GMFG,GPM6A,GPR158,HAPLN3,HBB,HBG1,HCN1,HHIP,HKDC1,HLA-DRA,HLAE,HLAJ,HMGA2,HNMT,HPGD,ICAM1,ICAM2,IGFBP5,IGHG1,IL11,IL13,IL1RAPL1,IL20RB,IL23A,IL6,IL7R,IRF6,ITGA7,ITPR1,JUN,KDM6B,KDR,KIR2DL1/KIR2DL3,KIT,KRT15,LEF1,LINC00589,LOXL2,MAFF,MAOB,MC4R,MEF2C,MEIS1,MELTF,MGP,MIR31HG,MMP1,MMP7,MT1H,MT1L,MUC1,MYH11,MYL4,MYLK,MYRF,NANOG,NCALD,NECTIN1,NFKBIZ,NIM1K,NMRAL2P,NOS3,NOSTRIN,NR2F2,NRIP3,NRP1,NTNG1,OCIAD2,PAEP,PCDH1,PDK1,PER3,PERP,PGM2L1,PHEX,PITX2,PLPP3,PON3,PPARG,PTHLH,PTX3,RAB27A,RAB38,RBP1,RETN,RGS2,RHBDL2,RRM2,S100A2,S100A3,SCG5,SELENOP,SEMA3B,SERPINB2,SERPINB5,SERPINB7,SERPINE2,SERPINH1,SFTPB,SFTPC,SHH,SLAMF9,SLC11A1,SMAD6,SOCS2,SOD3,SOX17,SOX2,SPHK1,SPINK1,SPP1,SPRY2,SRPX,STK33,STXBP6,TAL1,TBX2,TBX21,TCIM,TEC,TFF1,TGFB2,THY1,TM4SF19,TMEM117,TMEM171,TMEM204,TNFRSF10C,TNFRSF9,TNFSF13,TNNC2,TNNI2,TNNT2,TPK1,TREM1,TRIM15,TRIM36,TRNP1,TSPAN13,TSPAN8,TUBB,TWF1,TYMS,TYRP1,UBD,ULBP2,UNC13D,VIM,WNT7A,WT1
**GATA2**	Inhibited	ABCA13,ADGRE5,ADGRG1,AK1,ALAS2,ALOX5,AMY2B,ANGPT1,AQP9,AR,AZGP1,BPIFB1,C11orf97,CAMP,CCDC68,CCR8,CD177,CD34,CD36,CD69,CDH5,CEL,CELSR3,CEP126,CES1,CHGA,CHI3L1,CLDN18,CLEC4E,CLEC4F,CMA1,CPA3,CSTA,CTSG,CXCL10,CYBB,CYP2F1,CYP4F11,DENND2A,DIO2,DMBT1,E2F2,ECT2L,EDN1,EDNRA,ELANE,EMCN,ERG,ETS1,F2RL2,FABP4,FAT1,FCER1A,FCN1,FCRLA,FGD4,FOXP1,FYB2,GABRP,GATA1,GATA2,GBX2,GCSAM,GDA,GFI1B,GFRA1,GIMAP8,GP6,GP9,GPR65,GPX3,GRTP1,GUCA2A,HBA1/HBA2,HDC,HEMGN,HOXA10,HSD17B1,ICAM2,ICAM4,IL13,IL1RL1,IL3RA,IL6,INKA2,ITGA2,ITGAM,KDR,KEL,KHDC1L,KIT,KLF2,KRT13,KRT31,LMO2,LTB4R,LTF,LYPD6B,MAOB,MAT1A,MCEMP1,MEF2C,MEP1A,MFSD2B,MMRN1,MPIG6B,MPL,MRGPRX3,MS4A2,NFE2,NOS3,OLFM4,P2RX1,PGR,PPARG,PRSS1,PRSS3,RAB44,RGS13,RGS18,RXRG,S100A9,SAMD5,SELP,SERPINB2,SERPINI2,SKP2,SLC18A2,SLC35D3,SLC4A1,SLC51A,SNTG2,SOX18,SOX2,SPI1,SPN,TAL1,TFR2,TFRC,TGFBR3,TGM2,TMEM40,TMPRSS2,TNFSF4,TPSAB1/TPSB2,TPSG1,TREML1,TRIM58,TSPAN12,TSPAN32,TUBB1,TXK,UACA,UBXN10,UGT1A6,UGT2B7,ZNF750
**MEF2C**	Inhibited	ABRA,ACTC1,ACTN2,ATF3,BDNF,CACNA1C,CASQ2,CCBE1,CCL2,CKM,COL10A1,COL1A1,COL1A2,COL2A1,COL3A1,CXCL2,CXCL6,DES,ECM2,FAP,FOS,FOSB,HSPB7,IBSP,IL11,IL6,JUN,JUND,KCNA5,KLF2,MEF2C,MMP10,MMP13,MMP25,MMP8,MYH1,MYH6,MYL4,MYOM1,MYOM2,MYOT,MYOZ1,MYOZ2,NFATC2,NR4A1,PDLIM3,POSTN,PPARGC1A,PTGS2,PTH1R,PTPRB,RUNX2,RYR2,S100A4,SFRP2,SLC8A1,SOST,TNNC1,TNNI1,TNNI2,TNNT1,TNNT2,TTN,VIM,ZFP36
**ZBTB17**	Inhibited	ANLN,ASPM,AURKB,BUB1B,CAMK2N1,CCNA2,CCNB1,CCNB2,CDC20,CDCA8,CDK1,CDKN1C,CENPF,CEP55,CKAP2,DCT,ECT2,EDA2R,EGR1,FOXM1,HMMR,IQGAP3,JUN,KIF11,KIF18B,KIF20A,KIF22,KIF2C,KIF4A,KIT,KLK8,LDLR,LRRN3,MKI67,NCAPH,NDC80,NEK2,NGFR,NUF2,NUSAP1,PBK,PIMREG,PLK1,PMAIP1,PRC1,PSRC1,RACGAP1,SGO1,SGO2,SPTBN1,TOP2A,TPX2,UBE2C,ZFP36
**CEBPA**	Inhibited	A2M,ABCA3,ABCB1,ADH1A,ADH1B,ADH1C,ADH6,ADH7,ADRB3,AGT,AKAP12,AKR1B10,AKR1C1/AKR1C2,AKR1C3,ALOX5AP,ANPEP,APLN,APOB,AQP5,ARG1,ARG2,ARL4C,ASNS,BIK,CA2,CAMP,CCNA2,CCNB2,CD19,CDK4,CETP,CFD,CHI3L1,CKAP4,CNFN,COL10A1,COL1A1,COL1A2,CPB2,CRABP2,CSF1,CSF3,CSF3R,CYP2A6,CYP2B6,CYP3A5,CYP3A7,CYP7A1,DHCR7,DLX3,E2F1,EBF1,EEF1A2,ELANE,EMCN,EPHA7,F8,F

		ABP4,FCAR,FHL1,FOS,FOXA2,FOXM1,G0S2,GAL,GAPDH,GATA2,GATA6,GBX2,GFI1B,GGH,GLRX,GP9,GPR84,GRHL3,HAMP,HDAC1,HGF,HMGA1,HNF1A,HP,HPGD,HPR,ICAM1,ICAM2,IGFBP3,IGHE,IL1RN,IL6,IL6R,IRF6,ITGAL,ITGAM,IVL,JUN,KCNMB1,KLF4,KLK8,KRT14,KRT17,KRT27,KRT7,LEFTY1,LEP,LEPR,LHCGR,LHX5,LPL,LST1,LTF,MMP13,MMP8,MMRN1,MNDA,MSI2,MSX2,MYBL2,MYCN,NFATC2,NR1H4,NR4A2,NR5A2,NRP1,NTF4,OLR1,ONECUT1,OTC,OVOL1,PAX5,PCK1,PCNA,PFN2,PLOD2,PPARG,PPARGC1A,PPP1R14A,PPP1R3C,PTGFR,PTGS2,PTPRE,PTX3,QKI,RETN,RGS2,RUNX2,S100A9,SAA1,SBSN,SCGB1A1,SCGB3A2,SEMA3E,SERPINB2,SERPINB5,SFTPA1,SFTPB,SFTPC,SFTPD,SOCS3,SPI1,SPINT2,SPP1,SPRR1A,SPRR3,STAR,STEAP4,TAC1,TFAP2A,TFR2,TGFB2,THBD,THRB,TNFRSF19,TSC22D3,ZIC2,ZNF296
**CDKN1A**	Inhibited	ANLN,ASPM,ATAD2,AURKA,AURKB,BIRC5,BRCA1,BUB1,BUB1B,CCL2,CCN1,CCN2,CCNA2,CCNB1,CCND3,CCNE1,CDC20,CDC25A,CDC25C,CDC6,CDK1,CDK4,CDKN2A,CDKN3,CENPF,CEP55,CHAF1B,CHEK1,CIT,CKS1B,COL1A2,CPA3,DLGAP5,DTL,DUSP1,EDNRA,EXO1,FANCG,FANCI,FAT2,FOXM1,H2AFX,H2AFZ,HJURP,IL6,ITGAM,JAG1,KIF20A,KIF2C,KNSTRN,KNTC1,MAD2L1,MCM2,MCM3,MCM4,MCM6,MCM7,MKI67,MMP1,MMP3,MMP9,MUC2,MYBL2,NCAPD3,NUSAP1,ORC1,P4HA1,PBK,PCLAF,PCNA,PHGDH,PLK1,POLD1,PRC1,RACGAP1,RAD51,RFC4,RRM1,SAA1,SERPINI2,SMC2,SMC4,SPAG5,SPDL1,STMN1,SUV39H1,TERT,TGFA,TNFRSF18,TNFSF13,TOP2A,TPX2,TTK,TUBB3,TUBB4A,TYMS,UBE2C,UBE2S,UBE2T,VIM,WDHD1,WNT3

Table S2. Upstream regulator analysis of DEGs in lung ADC predicted by IPA.

Upstream Regulator	Prediction Activation State	Target Molecules in Dataset
**ERBB2**	Activated	ABCB1,ABCC8,ACVRL1,ADAMTS1,ADCY4,ADRA1B,ADRA1D,AFAP1L1,AGER,AGTR1,AKAP12,ALAS2,ALOX12,ALOX15B,ALOX5,ALPL,APOA1,AR,ATF3,BDNF,BIRC5,BMP7,CAD,CAMP,CAT,CAV1,CCL2,CCNA2,CD55,CD79B,CDC25C,CDH1,CDK1,CDKN2A,CDKN2B,CEACAM1,CES1,CHI3L1,CHRNA3,CHRNA5,CHRNB4,COL1A1,COL1A2,COL7A1,CR2,CRABP2,CRYAB,CRYBB2,CXCL12,CXCL3,CXCL5,CYBRD1,CYP11A1,CYP17A1,DBF4,DBH,DIAPH2,DLC1,DLX3,DMD,DRD1,E2F1,EDA,EGR1,ENG,EREG,F10,FGF10,FGF2,FGFR4,FOS,FOSL1,FOXF2,FOXM1,FOXP3,FPR2,GABRA4,GHR,GJA1,GP6,GRIA1,GRIN1,HAS1,HAS2,HAS2-AS1,HBB,HBEGF,HBG2,HDC,HGF,HMGA1,HNF4A,HSPA5,ID4,IGF2,IGFBP1,IGFBP3,IL1A,IL1B,IL21R,IRAK3,JAM2,JUN,KCNQ3,KDR,KISS1,KLF4,KLF6,KRT16,KRT19,KRT4,KRT81,LCAT,LDLR,LHB,LPL,LTB,MAOA,MAOB,MEOX2,mir-27,MMP1,MMP11,MMP14,MMP9,MPL,MUC4,MUC5AC,MUC5B,MYBL2,MYH11,MYLK,NCF2,NES,NFKBIA,NOS1,NPR1,PADI1,PADI4,PCK1,PDGFB,PDK1,PENK,PGR,PLAC1,PLAU,PMAIP1,PNMT,PRKCE,PRKG1,PROM1,PTCRA,PTGER4,PTGS2,PTH1R,PTN,PTTG1,RASGRP2,RECK,RECQL4,ROBO4,RRAS,RUNX2,SCTR,SERPINE2,SFTPA2,SGK1,SLC11A1,SLC2A1,SLC2A12,SLC2A3,SLC39A8,SLC5A1,SLC6A2,SPN,SPP1,SPTB,STAR,STX1A,TERT,TFF1,TGFB2,TGFBR2,TGFBR3,TGM1,THBD,THRB,TIMP1,TIMP3,TINCR,TNFAIP3,TNFSF11,TNFSF14,TNNC1,TP73,TYMS,UGDH,UGT1A7 (includes others),VIM,VWF,WNT9A,ZEB2

**RABL6**	Activated	AURKB,BIK,BTG2,BUB1,BUB1B,CCNA2,CCNB1,CCNE2,CDC25A,CDC25C,CENPF,CHEK1,CHEK2,CKS1B,CX3CL1,EZH2,FEN1,FERMT2,H2AFX,HBEGF,HMMR,KIF23,MAD2L1,MCM10,MCM2,MCM7,MELK,NCAPG,NDC80,NEK2,PBK,PLK1,PMAIP1,POLE2,PRC1,PTPRM,RAD54B,SMAD6,TOP2A,TPX2,TTK,UBE2C
**CCND1**	Activated	AHNAK,AR,AREG,ARMCX1,ASPM,ATAD2,ATP2B1AS1,AURKA,BIRC5,BRCA1,BRIP1,C7,CCNA1,CCNA2,CCNE1,CCNE2,CDC45,CDC6,CDCA2,CDCA7,CDCA8,CDH1,CDKN2A,CDKN2B,CENPF,CENPH,CENPK,CEP55,CIP2A,CLIC6,CLSPN,COL1A1,COL5A2,CPEB1,CPED1,CST4,DEPDC1,DKK2,DTL,E2F1,E2F7,E2F8,ENO1,EPGN,EREG,ESCO2,FAM83D,FOXM1,GPRC5A,GPRIN2,HIST2H2BE,HJURP,HSPB8,ID1,ID3,ITIH2,JPT1,KDM6B,KIF11,KIF20A,KIF2C,KIF4A,KLHDC1,KNL1,KRT1,KRT4,LINC00467,LRRTM2,MACF1,MAFF,MAGI2,MC4R,MCM10,MCM4,MCM7,MEIS2,MELK,MMP3,MYLK,MYO7A,MYRIP,NCAPH,PAH,PCLAF,PCNA,PCYT1B,PDLIM3,PGR,PLK1,PPARG,PRSS1,RAB3B,RACGAP1,RAD51,RBMS3,RGS2,RHPN2,RMI2,RRM2,SCG5,SELENBP1,SLC4A4,SOX2,SOX4,SPC25,SPP1,ST6GALNAC1,ST6GALNAC5,STOM,TCF4,TGFBR2,TMEM204,TMPRSS2,TMSB15A,TPX2,TRAIP,TRIP13,TRPM8,TUBB1,TYMS,WNT7A,ZNF423
**FOXM1**	Activated	ACE,ANXA1,AR,AURKB,AXIN2,BIRC5,BRIP1,BUB1B,CAT,CAV1,CCNA1,CCNA2,CCNB1,CCNB2,CCNE1,CCNE2,CCNF,CDC20,CDC25A,CDC25C,CDCA2,CDCA8,CDH1,CDK1,CDKN2A,CDKN3,CENPA,CENPE,CENPF,CKS1B,CKS2,CXCL12,CYP7A1,FOS,FOXF1,GTSE1,IL6,JUN,KDR,KIF20A,LAMA4,MCM8,MKI67,MMP7,MMP9,MUC5AC,NEK2,NES,PCNA,PLK1,PLK4,PRC1,PROM1,PTTG1,SFRP1,SFTPB,SFTPD,SOX2,TGFBR2,TOP2A,TWIST1,TWIST2,VIM,ZEB1,ZEB2
**PTGER2**	Activated	ADGRE1,AREG,ASPM,AURKA,BRCA1,BUB1B,CCNA2,CCNB2,CDH1,CDKN3,CENPE,CENPF,CEP55,CFP,CKAP2L,CKS2,CLEC4D,CLEC6A,CXCR2,DEPDC1,ECT2,EGR1,FOXP3,FPR1,H2AFX,HAMP,HDC,HIST1H2AD,IL1A,IL1B,IL23A,IL36G,IL6,KIF11,KIF15,KIF18B,KIF20A,KIF2C,MELK,MKI67,MMP9,NEK2,NOS2,NUF2,NUSAP1,PBK,PGR,PLK1,PRC1,PTGES,PTGS2,RACGAP1,SPAG5,SPP1,STAR,STIL,TIMP1,TIMP3,TPX2,TREM1,TROAP,TTK
**SOX2**	Activated	ABCC3,ABCG2,ADAMTS4,ALDH1A1,ALOX5,ANGPT1,APCDD1,AQP4,ARHGEF10,ASCL2,ASPRV1,ATF3,AXIN2,BFSP2,BIRC5,BMPER,C1QL1,CASKIN2,CCN4,CCN5,CDH1,CDKN2A,CITED1,CLDN11,CNFN,COL1A1,COL9A3,CRABP2,CTNNAL1,CXCL14,CYP2J2,DDC,DKK3,DLL1,DLX1,DLX3,DLX4,DLX5,DNMT3B,DSP,DUSP1,EFEMP1,EPB41L2,EPCAM,ETS2,ETV4,FA2H,FABP7,FGFR4,FOS,FOXA2,FOXD3,FOXE1,FOXF1,FOXH1,GAB1,GADD45B,GATA2,GATA6,GBP4,GCK,GJA1,GJC2,GPR17,GRHL3,H19,HOXC4,ID2,ID3,IL23A,INAVA,JAM3,JUN,KDR,KIF26A,KLF4,KLF6,KRT19,KRT27,KRT6A,LHX2,LIMS2,LMCD1,LRRC4,MAG,MEIS1,MFSD2A,MIXL1,MTMR10,MYRF,NFASC,NOTCH4,NR0B1,OLIG1,ONECUT1,OTX1,OVOL1,PCDH15,PDK1,PEG3,PIK3R1,PITX2,PLAC1,PLLP,PLP1,PLXNB3,POU4F1,PPARG,PRKCQ,PROM1,PSAT1,RASGEF1B,RNF43,S1PR5,SALL4,SAPCD2,SEMA3D,SEMA5A,SEMA6A,SGK2,SNX22,SOX10,SOX13,SOX17,SOX2,SPRR3,SPRY4,STRA6,TBX3,TCL1A,TGFB2,TGM1,TIMP3,TMEM100,TNNI1,TNR,TSPAN2,TUBB3,TWIST1,TWIST2,UGT8,UST,VIM,WLS,WNT3,ZFP42,ZFP57,ZIC2
**FGF2**	Activated	ACAN,ACE,ADGRL2,AGTR2,AKR1B10,ANGPT1,ANPEP,AR,ARC,AREG,AXIN2,BDNF,BIRC5,BTG2,C1QL1,CAV1,CCK,CCL2,CCN1,CCN3,CCNE1,CDC25A,CDH1,CHRM3,CHRM4,CNMD,COL1A1,COL1A2,COL3A1,CRYAB,CSF3,CXCL12,CXCL2,CYGB,CYP11A1,DCN,DIO3,DKK2,DLL1,EDNRB,EFNB2,EGLN3,EGR1,ELN,ENO1,EPAS1,EPCAM,EREG,ETV1,ETV4,FABP4,FGF2,FGFR2,FGFR4,FOS,FOSL1,FUT2,FUT3,GDNF,GFAP,GJA1,G

		REM1,GRIA1,GRIA3,GRIK4,GRIN2A,HAS2,HBEGF,HGF,HTR3A,IBSP,ID2,ID3,IFNE,IGF2,IGFBP3,IL1B,IL6,ITGA1,JUN,JUNB,KDR,KL,LYVE1,MEOX2,MGP,MIF,MITF,MKI67,MMP1,MMP13,MMP3,MMP9,MSX1,MUC5AC,NES,NKD1,NOS2,NOTCH4,NR3C2,NR4A1,NR4A2,PCNA,PCSK2,PDGFB,PENK,PITX2,PLAU,PPARG,PRKCE,PRSS50,PTGS2,PTTG1,RUNX2,S100A4,S100A8,SDC1,SDC2,SELE,SEMA6D,SFRP1,SHH,SLC2A1,SLC34A1,SOCS3,SOX17,SPINK1,SPP1,SPRY4,STAR,STOM,TAC1,TAT,TFPI,TGFB2,TGFBR3,TIMP1,TIMP3,TNFSF11,TOP2A,TSHR,TUBB3,TWIST1,TWIST2,UGT2B15,UGT2B17,VGF,VIM,VWF,WNT3A,ZEB1,ZEB2,ZFP57
**IL1B**	Activated	A2M,ABCC3,ABCG2,ACAN,ACHE,ADAM8,ADAMTS1,ADAMTS4,ADRB2,AGER,AGTR1,ALOX15,ALPL,ANGPT1,ANXA1,ANXA9,AQP4,ARC,ARG1,ATF3,BMP2,BTG2,CALCA,CAT,CCL2,CCL24,CCL7,CCR8,CCRL2,CD274,CD55,CD69,CD83,CDH22,CDX1,CFB,CFTR,CHI3L1,CHST6,CNR2,COL10A1,COL1A1,CP,CPB2,CRYAB,CSF3,CSRNP1,CX3CL1,CX3CR1,CXCL12,CXCL13,CXCL2,CXCL3,CXCL5,CXCL9,CXCR1,CYP11A1,CYP1A1,CYP1A2,CYP27B1,CYP3A5,CYP7A1,DCN,DUSP1,E2F1,E2F2,EDN1,EGLN3,EGR1,EIF4EBP1,ELN,ENG,EPAS1,ETS2,FABP5,FGB,FGF18,FGF2,FGFR2,FOS,FOSB,FOSL1,FPR2,GAD1,GADD45B,GAS6,GCK,GCLC,GHR,GJA1,GRB7,GRIA1,H19,HAMP,HAS1,HAS2,HAS2AS1,HBEGF,HGF,HLAE,HMGA1,HNF4A,HOTAIR,HP,HPGDS,HPRT1,HSPA5,IBSP,ID3,IGFALS,IGFBP1,IGFBP3,IGFBP6,IL11,IL16,IL18R1,IL18RAP,IL1A,IL1B,IL1RL1,IL23A,IL2RA,IL33,IL36RN,IL3RA,IL4I1,IL6,IRAK1,IRAK3,ITGA1,JUN,JUNB,JUND,KDR,KIF15,KLF10,KRT19,LAMA3,LBP,LCN2,LDLR,LEP,LHB,LIFR,LTA,MAP3K8,MARCKSL1,MEF2B,MEFV,MFAP2,MIF,MMP1,MMP10,MMP11,MMP13,MMP14,MMP3,MMP7,MMP9,MT2A,MUC3A,MUC4,MUC5AC,MUC5B,MYH11,MYLK,NFKBIA,NFKBIZ,NLRP7,NOS1,NOS2,NOX1,NQO1,NR0B2,NR1H4,NR4A1,NR4A2,NR4A3,OLR1,P4HB,PCNA,PCSK1,PCSK2,PDGFB,PDK1,PENK,PFKP,PIGR,PLA2G2A,PLA2G3,PLA2G5,PLAU,PLK3,POSTN,PPARG,PPARGC1A,PPARGC1B,PPP1R14A,PTGDS,PTGES,PTGFR,PTGIS,PTGS2,PTPRN,PTX3,RAB3C,RCAN1,RHOB,RORA,RUNX2,S100A8,SAA1,SAA2,SAA2SAA4,SCNN1B,SCNN1G,SCUBE1,SDC1,SELE,SELENOP,SERPINB2,SESN1,SFTPA1,SGPL1,SHH,SLC14A1,SLC1A2,SLC25A25,SLC2A1,SLC2A4,SLC6A4,SLC7A11,SNCA,SOCS2,SOCS3,SPP1,STAR,STMN2,SULT1E1,TAC1,TACR1,TERC,TFF1,TGFBR2,TGM1,THBD,THY1,TIMP1,TIMP3,TINCR,TK1,TLR3,TLR4,TLR8,TNFAIP3,TNFRSF9,TNFSF11,TREM1,TSC22D3,TSLP,TWIST1,UBD,UGDH,UGT1A1,VIM,VSNL1,XDH,ZFP36
**SP1**	Activated	ABCB1,ABCC8,ACVRL1,ADAMTS1,ADCY4,ADRA1B,ADRA1D,AFAP1L1,AGER,AGTR1,AKAP12,ALAS2,ALOX12,ALOX15B,ALOX5,ALPL,APOA1,AR,ATF3,BDNF,BIRC5,BMP7,CAD,CAMP,CAT,CAV1,CCL2,CCNA2,CD55,CD79B,CDC25C,CDH1,CDK1,CDKN2A,CDKN2B,CEACAM1,CES1,CHI3L1,CHRNA3,CHRNA5,CHRNB4,COL1A1,COL1A2,COL7A1,CR2,CRABP2,CRYAB,CRYBB2,CXCL12,CXCL3,CXCL5,CYBRD1,CYP11A1,CYP17A1,DBF4,DBH,DIAPH2,DLC1,DLX3,DMD,DRD1,E2F1,EDA,EGR1,ENG,EREG,F10,FGF10,FGF2,FGFR4,FOS,FOSL1,FOXF2,FOXM1,FOXP3,FPR2,GABRA4,GHR,GJA1,GP6,GRIA1,GRIN1,HAS1,HAS2,HAS2AS1,HBB,HBEGF,HBG2,HDC,HGF,HMGA1,HNF4A,HSPA5,ID4,IGF2,IGFBP1,IGFBP3,IL1A,IL1B,IL21R,IRAK3,JAM2,JUN,KCNQ3,KDR,KISS1,KLF4,KLF6,KRT16,KRT19,KRT4,KRT81,LCAT,LDLR,LHB,LPL,LTB,MAOA,MAOB,MEOX2,mir27,MMP1,MMP11,MMP14,MMP9,MPL,MUC4,MUC5AC,MUC5B,MYBL2,MYH11,MYLK,NCF2,NES,NFKBIA,NOS1,NPR1,PADI1,PADI4,PCK1,PDGFB,PDK1,PENK,PGR,PLAC1,PLAU,PMAIP1,PNMT,PRKCE,PRKG1,PROM1,PTCRA,PTGER4,PTGS2,PTH1R,PTN,PTTG1,RASGRP2,RECK,RECQL4,ROBO4,RRAS,RUNX2,SCTR,SERPINE2,SFTPA2,SGK1,SLC11A1,SLC2A1,SLC2A12,SLC2A3,SLC39A8,SLC5A1,SLC6A2,SPN,SPP1,SPTB,STAR,STX1A,TERT,TFF1,TGFB2,TGFBR2,TGFBR3,TGM1,THBD,THRB,TIMP1,TIMP3,TINCR,TNFAIP3,TNFSF11,TNFSF14,TNNC1,TP73,TYMS,UGDH,UGT1A7 (includes others),VIM,VWF,WNT9A,ZEB2

**HDAC1**	Activated	ABCB1,ACAN,AKAP12,APOA1,AR,ARC,ASCL2,ATF3,AXIN2,BDNF,CAVIN2,CCNA1,CCNA2,CCNB1,CCNB2,CCNE1,CCNE2,CCR8,CD27,CD34,CDC25A,CDC25C,CDC6,CDH1,CDK1,CDKN2A,CDT1,COL1A1,COL1A2,COL9A1,DUSP1,E2F2,EGR1,EPGN,FABP4,FAM107A,FOS,FOXM1,FOXP3,GABARAPL1,H2AFX,HBG1,HBG2,ID1,IL13,IL6,INA,KDR,KRT15,LCN2,LGR5,LMO2,MCM10,MCM7,MME,MMP9,MT1G,MUC4,MYBL2,NFKBIA,NKD1,NOS2,OLFM4,OSCAR,PCK1,PDGFB,PLAU,PLK1,PMAIP1,PPARG,PPARGC1A,PTGS2,RAD54L,RECK,RECQL4,RHOB,RRM2,RUNX2,SCN2A,SERPINB2,SFRP1,SGK1,SLC1A2,SOX10,SOX2,SPP1,STX1A,SYT4,TAL1,TBX2,TBX5,TERT,TGFBR2,TNNI2,TNS1,TOP2A,TP73,TPD52,TSC22D3,TUBB3,TYMS
**VEGF**	Activated	A2M,ABCB1,ACAN,ACE,ACKR4,ADAMTS1,ADGRF5,ADGRL2,AKAP12,ALOX5AP,ANGPT4,ANGPTL7,ANPEP,APLN,AQP4,ATF3,AURKA,AURKB,AXIN2,BIRC5,BMP2,BMP7,BNC1,BTN1A1,BUB1,BUB1B,CA2,CALCRL,CCL7,CCN1,CCNE2,CCNF,CD3EAP,CD93,CDC14A,CDC20,CDC25A,CDC25C,CDC45,CDC6,CDH1,CDH5,CDK1,CDKN2A,CDKN3,CENPF,CHI3L1,CHIA,CKS1B,CNN1,CNTFR,COL4A3,CPA3,CRABP1,CRTAC1,CRYAB,CXCL12,CXCR2,CYP4A11,CYYR1,DBF4,DDX11,DIAPH2,DOK5,DTYMK,EDA,EDN1,EFNB2,EGR1,EGR3,ELN,EMCN,EMP1,EMP2,EPHX4,FABP4,FGF2,FLT4,FOSB,FOSL1,FOXM1,FXYD4,GAB1,GATA1,GJB2,GMNN,GPR4,GRK5,HBEGF,HDC,HELLS,HMGCS2,HMMR,ID1,IGFBP3,IHH,IL11,IL1A,IL3RA,IL5RA,IL6,INHBB,ITGA10,JAM2,JUN,KDR,KIF11,KIF15,KIF2C,LCN2,LDLR,LRAT,LRP2,LYVE1,MAD2L1,MAGI1,MAOB,MAP3K8,MCM2,MELK,MEOX2,MGP,MIF,MKI67,MMP10,MMP13,MMP14,MMP9,MT1G,NDC80,NEK2,NLRP12,NOSTRIN,NOTCH4,NR4A1,NR4A2,NR4A3,NRG1,P2RY14,PADI1,PC,PCDH10,PDE7B,PHLDA2,PIM2,PKDCC,PKMYT1,PLAU,PLK1,PLK4,PLOD2,PLPP3,PLXNA2,PMAIP1,PMCH,PMP22,PRC1,PTGS2,PTPN5,PTPRR,RAMP3,RCAN1,RCC1,RECK,RGCC,RGS2,RGS20,RGS9,RNF125,SELE,SERPINB2,SFRP1,SGK1,SH3TC2,SHISA2,SNRK,SOCS2,SOCS3,SSTR1,STIL,TACR1,TGFBR2,THBD,TIMP1,TLR3,TMEM158,TNFRSF9,TPX2,TRAIP,TRIP13,TRPC4,TTK,UBE2C,VIM,VWF,XDH,ZEB1
**CG**	Inhibited	ABCB1,ADAMTS1,ADCYAP1,ADCYAP1R1,AKR1B10,APCDD1,APLN,AR,AREG,BDNF,BLM,BMP2,BTG2,CCL2,CCNE2,CD93,CDC6,CDH1,CLDN11,CLU,COL11A1,CTSV,CXCL3,CYP11A1,CYP17A1,DMD,DPT,DTL,DUSP1,EDN2,EDN3,EFEMP1,EFNB2,EGR1,EMCN,EMP1,EPAS1,EPHA3,EPHA7,EPHB6,EREG,FABP4,FABP5,FAM3D,FGF9,FHL1,FOS,FZD4,GAL,GAS1,GATA6,GGCT,GJA1,GJB2,HAS2,HMGA1,HMGA2,HPGD,HSD17B3,IGFBP3,IL11,IL1B,IL33,IL6,INHA,INHBB,ITGA1,JUN,KCNJ3,KCNT2,KISS1,KLF4,LDLR,LEPR,LIMCH1,LRP1B,LRRN3,MCM10,MMP1,MMP19,MMP9,NOS2,NPR1,NPR3,NR2F1,NR3C2,NR4A1,PCNA,PDE4D,PGR,PKIA,PLAU,PLPP3,PMAIP1,PNOC,PODXL,PPARG,PPP1R14A,PRKG2,PTGFR,PTGS2,PTX3,RASSF2,RETN,RGS2,RGS20,RORA,RUNX2,SBSN,SCUBE1,SDC1,SEMA3E,SLC4A4,SLC52A2,SLC5A5,SLC5A7,SMAD6,SPP1,ST8SIA1,STAR,STEAP1,TAC1,TDRD5,TENM4,TGFB2,TGFBR3,TIMP1,TMEM158,TSHR
**CTNNB1**	Inhibited	ABCB1,ACTC1,ADGRV1,ADRA2C,ALDH1A1,ALDH1A2,ALG3,ANXA1,AOC3,APCDD1,AQP4,AR,ASCL2,AURKA,AXIN2,BCL2L10,BIRC5,BMP2,BMP7,C2orf40,C6,CA3,CADM1,CCL7,CCN1,CCN4,CCNA1,CCNA2,CCNE1,CCNE2,CD34,CD36,CDH1,CDH16,CDKN2A,CDKN2B,CDX1,CEACAM1,CENPM,CFD,CHEK2,CLDN2,CLDN5,CLU,CNMD,CNR1,CNTFR,COL1A1,COL4A5,COL4A6,CRABP2,CRIP1,CSF3,CST4,CTHRC1,CTNND2,CXCL12,CXCL2,CYP11A1,CYP1A1,CYP1A2,CYP24A1,CYP4F12,DBH,DCT,DES,DIO2,DIXDC1,DPEP1,EDIL3,EDN1,EDN3,EGR1,EPCAM,EPHA5,EPHB2,EPHB3,ETV1,ETV4,FABP4,FCAMR,FCRL5,FEN1,FGF18,FGF9,FOS,FOSL1,FOXA2,FOXC2,FSTL3,FUT2,GAD1,GADD45B,GATA2,GDNF,GFAP,GHR,GJA1,GJB2,GPA33,GPX2,GREM1,GRHL2,GRIK1,HHIP,HILPDA,HMGCS2,HNF1A,HNF4A,HOXA5,HOXB7,ID1,ID2,ID3,ID4,IGF2,IGF2BP1,IGHM,IHH,IL13,IL1B,IL23A,IL2RA,INHA,INHBB,ITGA1,JAM2,JUN,KDR,KIF23,KIFC1,KRT1,LGR5,LHB,LHX2,LHX6,LMO2,LPL,MEOX1,MITF,MME,MMP1,MMP13,MMP14,MMP3,MMP7,MMP9,MSX1,MT1L,MUC6,MYBPC1,MYH6,MYLK,NCAM1,NDRG2,NES,NKD1,NOS2,NOTUM,NOX1,

		NR0B1,NR4A1,NRG1,OGN,OLIG1,OR51I1,PCDH15,PCDH9,PCNA,PDE1C,PEG3,PHLDA2,PIK3R1,PIP5K1B,PITX2,PKP3,PLAU,PLK1,PMCH,PMP22,POU3F2,PPARG,PRSS35,PRX,PTCRA,PTGS2,QPCT,RBP4,RETN,RGN,RUNX2,S100A4,S100A8,SAA1,SDC2,SEMA5A,SERPINE2,SESN1,SFRP1,SGK1,SHH,SHOX2,SIM2,SIX1,SLC17A9,SLC1A2,SLC6A2,SMAD6,SORBS3,SOX11,SOX17,SOX2,SOX4,SOX5,SOX7,SP5,SPI1,SPP1,SPRY4,SRPK1,SYNM,TBX5,TCF4,TCL1A,TERT,TFF1,TGFA,TGM1,TIMP1,TIMP3,TLE4,TMSB15A,TNFRSF18,TNFSF11,TNNC1,TP73,TRAF4,TUBB2B,TWIST1,UPB1,VIM,WIF1,WNT11,WNT3,WNT3A,WNT6,ZEB1,ZEB2,ZFP42,ZIC1,ZP3
**SMARCA4**	Inhibited	A2M,ABCB1,ABLIM3,ACE,AGMAT,AGR2,AHNAK,AIM2,ALDH2,APOA1,APOL3,AREG,ARRDC4,AXIN2,BIRC5,CALB2,CARD16,CARD17,CCDC169,CCL2,CCNA2,CCNE2,CCR8,CCRL2,CD19,CD52,CDC25A,CDC6,CDH1,CDH16,CDKN2A,CDKN2B,CFP,CHEK2,CKM,CLIC2,CNTN1,COL1A1,COL7A1,CORO6,CP,CTSH,CXCR5,CYP1A1,CYP4B1,DCT,DES,DGKG,DLL1,DUSP13,E2F1,EBF1,ECM2,EDNRA,EFNB1,EGR1,EPHB2,EREG,ESPNL,ETS2,FABP4,FAM167A,FBP1,FFAR2,FGF9,FGFR2,FHL2,FMO2,FOLR1,FOS,FOXA2,FUT1,GADD45B,GAPDH,GCAT,GCLC,GJB5,GPM6A,HBB,HBG1,HHIP,HKDC1,HLA-E,HMGA2,HNF4A,HPGD,ICAM2,ID3,IGHG1,IL11,IL13,IL1B,IL1RAPL1,IL23A,IL6,IL7R,JUN,KCNQ3,KDM6B,KDR,KIR2DL1/KIR2DL3,KRT15,LAMA3,LINC00589,LOC728485,LRAT,MAFF,MAOB,MC4R,MEIS1,MELTF,MGP,MIR31HG,MIR503HG,MMP1,MMP7,MT1L,MYBPH,MYH11,MYLK,MYRF,NCALD,NECTIN1,NFKBIZ,NIM1K,NOSTRIN,NRIP3,NTNG1,OCIAD2,PAEP,PCSK9,PDK1,PGM2L1,PHEX,PHLDA2,PITX2,PKIA,PLPP3,PMEL,PPARG,PTX3,RAMP1,RETN,RGS2,RHBDL2,RRM2,S100A2,S100A3,SCG5,SDC2,SELENOP,SEMA3B,SERPINB2,SERPINB5,SERPINE2,SERTAD1,SFTPB,SFTPC,SHH,SLAMF9,SLC11A1,SLC2A4,SMAD6,SMIM10L2B,SOCS2,SOD3,SOX17,SOX2,SPINK1,SPP1,SRPX,STXBP6,TAL1,TBX15,TBX2,TCF4,TFF1,TGFB2,THY1,TMEM171,TMEM204,TMPRSS13,TNFRSF9,TNNI2,TNNT2,TNNT3,TREM1,TRIM15,TYMS,TYRP1,UBD,VIM,WNT3A,WNT7A,WNT9A
**GATA2**	Inhibited	ADGRE5,AK1,ALAS2,ALOX5,AMY2B,ANGPT1,AQP9,AR,C11orf97,CAMP,CCDC68,CCN3,CCR8,CD34,CD36,CD69,CDH5,CELSR3,CES1,CHI3L1,CLDN18,CLEC4E,CLEC4F,CMA1,CPA3,CTSG,CYP11A1,CYP2F1,DDIT4L,DENND2A,DIO2,DLL1,DMBT1,E2F2,ECT2L,EDN1,EDNRA,EMCN,EPHA3,ERG,FABP4,FCER1A,FCN1,FCRLA,FYB2,GATA1,GATA2,GFI1B,GFRA1,GIMAP8,GP6,GP9,GPX3,GRTP1,GUCA2A,HBA1/HBA2,HDC,HEMGN,HNF4A,HOXA10,ICAM2,ICAM4,IL13,IL1RL1,IL3RA,IL6,INKA2,KDR,KLF2,KRT13,LCN2,LMO2,LTF,LYPD6B,MAOB,MAT1A,MCEMP1,MEP1A,MFSD2B,MGST1,MMRN1,MPIG6B,MPL,MS4A2,OLFM4,P2RX1,PBX1,PGR,PPARG,PRSS1,PRSS3,RAB44,RCAN1,RGS13,RHCE/RHD,RXRG,S100A8,SAMD5,SELP,SERPINB10,SERPINB2,SERPINI2,SLC18A2,SLC4A1,SLC51A,SNCA,SNTG2,SOX18,SOX2,SPI1,SPN,TAL1,TFR2,TGFBR3,TMPRSS2,TNFSF4,TPSAB1/TPSB2,TPSG1,TRIM58,TSPAN12,TSPAN32,TUBB1,UACA,UGT1A6,ZNF365
**MEF2C**	Inhibited	ABRA,ACTC1,ACTN2,ATF3,BDNF,BLK,CASQ2,CCBE1,CCL2,CKM,CLSTN2,COL10A1,COL1A1,COL1A2,COL3A1,CXCL2,DES,ECM2,EGR2,FAP,FOS,FOSB,GJA1,HSPB7,IBSP,IL11,IL6,JUN,JUNB,JUND,KCNA5,KLF2,MMP10,MMP13,MMP25,MYH1,MYH6,MYOM1,MYOM2,MYOT,MYOZ1,MYOZ2,NR4A1,PDLIM3,PHKA1,PLA2G2A,PLAGL1,POSTN,PPARGC1A,PTGS2,PTH1R,PTPRB,RUNX2,RYR2,S100A4,SLC2A4,SRPK3,TNNC1,TNNI1,TNNI2,TNNT1,TNNT2,TTN,VIM,ZFP36

**ZBTB17**	Inhibited	ANLN,ASPM,AURKB,BUB1B,CAMK2N1,CCNA2,CCNB1,CCNB2,CDC20,CDCA8,CDK1,CDKN1C,CDKN2B,CENPF,CEP55,DCT,ECT2,EDA2R,EGR1,FOXM1,HMMR,IQGAP3,JUN,KIF11,KIF18B,KIF20A,KIF2C,KIF4A,KLK8,LDLR,LRRN3,MKI67,NCAPH,NDC80,NEK2,NUF2,NUSAP1,PBK,PIMREG,PLK1,PMAIP1,PRC1,RACGAP1,SGO1,SGO2,SPTBN1,TOP2A,TPX2,UBE2C,ZFP36
**CEBPA**	Inhibited	A2M,ABCA3,ABCB1,ADH1A,ADH1B,AKAP12,AKR1B10,ALOX5AP,ANPEP,ANXA1,APCDD1,APLN,ARG1,ASPRV1,BIK,BTG2,CA2,CAMP,CCNA2,CCNB2,CD19,CDH1,CFD,CHI3L1,CNFN,COL10A1,COL1A1,COL1A2,CPB2,CRABP2,CSF3,CSF3R,CYP11A1,CYP3A5,CYP3A7,CYP7A1,DLX3,E2F1,EBF1,EEF1A2,EFNB2,EGR2,EMCN,EPHA7,F8,FABP4,FCAR,FHL1,FOS,FOXA2,FOXM1,GAL,GAPDH,GAS1,GATA2,GATA6,GFI1B,GGH,GJA1,GP9,GPR84,GRHL3,HAMP,HAS2,HCAR3,HGF,HMGA1,HNF1A,HP,HPGD,HPR,HSPA5,ICAM2,ID1,ID2,IGFBP3,IGHE,IL6,IVL,JUN,JUNB,KLF4,KLK8,KRT27,LCN2,LEP,LEPR,LPL,LST1,LTF,MMP13,MMRN1,MNDA,MSI2,MT2A,MYBL2,NR1H4,NR4A2,OLR1,ONECUT1,OTC,OVOL1,PAX5,PCK1,PCNA,PFN2,PLOD2,PPARG,PPARGC1A,PPP1R14A,PPP1R3C,PTGFR,PTGS2,PTX3,RETN,RGS2,RORA,RUNX2,S100A8,SAA1,SBSN,SCGB1A1,SCGB3A2,SEMA3E,SERPINB2,SERPINB5,SFTPA1,SFTPB,SFTPC,SFTPD,SLC2A4,SOCS3,SPI1,SPINT2,SPP1,SPRR3,STAR,STEAP4,TAC1,TFAP2A,TFR2,TGFB2,THBD,THRB,TRAF4,TSC22D3,ZIC2
**CDKN1A**	Inhibited	ANLN,ASPM,ATAD2,AURKA,AURKB,BIRC5,BRCA1,BUB1,BUB1B,CCL2,CCN1,CCNA2,CCNB1,CCNE1,CDC20,CDC25A,CDC25C,CDC6,CDK1,CDKN2A,CDKN2B,CDKN3,CENPF,CEP55,CHAF1B,CHEK1,CIT,CKS1B,COL1A2,CPA3,CRYAB,DLGAP5,DTL,DUSP1,EDNRA,EXO1,FANCI,FAT2,FOXM1,H2AFX,HJURP,HSPA5,IL1B,IL6,KIF20A,KIF2C,KNTC1,MAD2L1,MCM2,MCM4,MCM6,MCM7,MKI67,MMP1,MMP3,MMP9,MSX1,MYBL2,NUSAP1,ORC1,PBK,PCLAF,PCNA,PLK1,PRC1,RACGAP1,RAD51,RFC4,SAA1,SERPINI2,SPAG5,TERT,TGFA,TGM1,TNFRSF18,TOP2A,TPX2,TRDN,TTK,TUBB3,TUBB4A,TYMS,UBE2C,UBE2S,UBE2T,VIM,WDHD1,WNT3

© 2020 by the authors. Licensee MDPI, Basel, Switzerland. This article is an open access article distributed under the terms and conditions of the Creative Commons Attribution (CC BY) license (http://creativecommons.org/licenses/by/4.0/).
